# Research on Error Compensation Methods of Dynamic Gravity Measurement Based on Swarm Cooperation Evolution Strategy and Optimized LSTM

**DOI:** 10.3390/e28050568

**Published:** 2026-05-19

**Authors:** Xinyu Li, Zhaofa Zhou, Zhili Zhang, Zhe Liang, Zhenjun Chang, Yiyi Li

**Affiliations:** College of Missile Engineering, Rocket Force University of Engineering, Xi’an 710025, China

**Keywords:** gravity measurement, dynamic error compensation, swarm cooperation evolution strategy (SCES), long short-term memory (LSTM), neural architecture search (NAS)

## Abstract

Dynamic gravity measurement, crucial for engineering application, suffers accuracy degradation due to errors induced by carrier maneuvers. To address the limitations of conventional swarm optimization algorithms in precision, stability, and generalizability, this study proposes a Swarm Cooperation Evolution Strategy (SCES) that efficiently integrates multiple algorithms. The proposed SCES is extensively evaluated on the CEC2022 benchmark suite in comparison with several cooperative fusion-related algorithms and representative single optimization algorithms. The experimental results demonstrate that SCES achieves an overall effectiveness score of 0.034 and an optimal accessibility rate exceeding 95%. Compared to the best-performing fusion-based algorithm, these metrics represent improvements of 54.67% and 31.11%, respectively. Moreover, relative to the best-performing single optimization algorithm, the improvements amount to 37.73% and 32.69%, respectively. These findings robustly validate the superior performance of the proposed algorithm. Moreover, an in-depth investigation based on SCES into dynamic error compensation methodologies is conducted. Firstly, a polynomial compensation model is established through error mechanism analysis, with parameters identified via SCES. Secondly, a data-driven compensation model employing a multi-layer long short-term memory (LSTM) network optimized via neural architecture search (NAS) guided by SCES is proposed, circumventing the performance limitations inherent in manually designed networks. Furthermore, an innovative two-stage hybrid strategy is introduced. Systematic trend errors are compensated using the polynomial model, followed by the NAS-LSTM model addressing complex residual nonlinear errors, effectively combining mechanism-based and data-driven approaches. Validation on three lines exhibiting varying maneuverability shows all methods significantly improve accuracy. The hybrid strategy delivers optimal performance, achieving 0.58 mGal internal coincidence accuracy on stable lines and up to 91.58% improvement in external coincidence accuracy under high maneuverability. This research provides an effective high-precision dynamic gravity measurement and compensation solution, advancing engineering applications.

## 1. Introduction

**Figure 1:**
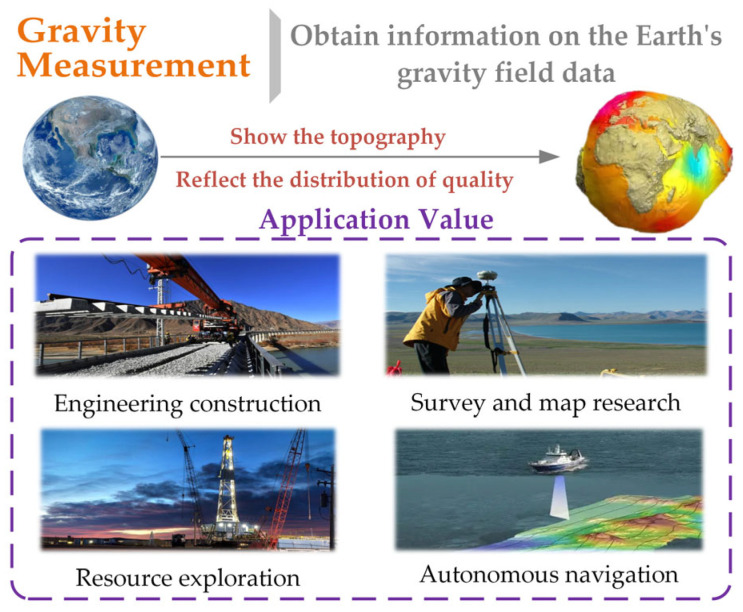
The Earth’s gravity field and its wide applications.

Precise gravity field data constitute a national strategic resource, and dynamic gravity measurement can rely on the carrier to quickly, efficiently, and accurately obtain the gravity information in the region. With the rapid development of artificial intelligence, dynamic gravimetry has emerged as a pivotal information medium for nations seeking to secure strategic advantages and establish technological preeminence [1,2,3]. As illustrated in Figure 1, the Earth’s gravity field not only reveals topographic and geomorphic features but also reflects the planet’s kinematic patterns and internal mass distribution. Dynamic gravimetry is dedicated to the rapid, efficient, and precise determination of regional gravity field information using mobile platforms. This endeavor holds considerable significance across diverse domains, including economic development, fundamental research, societal progress, and national defense security. On the one hand, gravity data facilitate the accurate inversion of mineral and energy resources—such as hydrocarbon reservoirs and geothermal deposits—advance foundational investigations in geophysics and geodesy, and support infrastructure engineering as well as seismic monitoring [4,5]. On the other hand, the gravity information also supports the development of industries 57 such as high-precision autonomous navigation, autonomous driving, and precision 58 instrument manufacturing, providing them with information services [6,7,8].

Nevertheless, measurement errors directly affect the practical application efficiency of gravity information. Dynamic errors under high-maneuverability conditions are particularly complex and pronounced, making the effective control and precise compensation of these errors a critical technological challenge requiring urgent breakthroughs [9,10]. Significant efforts have been devoted to suppressing dynamic measurement errors for enhanced precision. David B. et al. from the Technical University of Munich proposed a theoretical correction model for long-term drift errors in strapdown gravimetry, achieving a post-compensation accuracy of 2 mGal [11], and Cai et al. analyzed error mechanisms in strapdown gravimetry and proposed a generalized compensation model with parameters optimized via correlation analysis, leading to effective error suppression [12]. The application of these two compensation methods to platform-based gravimetry suffers from limited effectiveness. Li X. P. derived an exact tilt error compensation model for inertially stabilized platforms, effectively reducing short-wavelength random errors [13], and Pan et al. presented a method for compensating unmodeled errors by comparing actual navigation data with trajectory generator outputs, improving internal consistency accuracy by 19.6% [14], but the errors caused by carrier motion remain a persistent challenge. Huang et al. introduced a universal compensation model for residual errors from dynamic environmental effects, using mutual correlation analysis based on information criteria to suppress environmental noise during high-dynamic surveys. Nevertheless, the accuracy of parameter identification achieved through correlation analysis is still less than ideal [15]. Huang C. F. et al. implemented a Kalman filtering approach for atomic gravimeters, achieving post-processed residuals of 2.03 mGal [16]. Li X. Y. et al. constructed a long short-term memory (LSTM)-based error compensation model for vehicle-mounted dynamic measurements, validating its feasibility under both stable and high-dynamic conditions [17]. However, the application of this machine learning-based compensation method is limited by the absence of a solid theoretical foundation.

In the field of measurement error compensation, constructing error models and accurately identifying their parameters represents the most prevalent and intuitive methodology, where swarm intelligence algorithms serve as effective tools for optimal model identification. Li X. Y. et al. employed an improved Sparrow Search Algorithm (SSA) and the alternating direction method of multipliers to fit temperature and vibration error models for gravimeters, effectively suppressing measurement errors [18,19]. Li X. et al. optimized vibration transfer function parameters using Particle Swarm Optimization (PSO), GA, and fruit fly optimization, achieving vibration error suppression with an accuracy of 2 μGal in laser interferometric gravimeters [20]. Wu Y. X. developed a coupled error model for shipborne gravimeters incorporating vertical fluctuations, sway, and horizontal accelerations. By optimizing transfer function parameters via PSO, a system accuracy of 1.1 mGal was attained [21]. However, while conventional swarm intelligence optimization algorithms and their derivative improvement strategies employed in these studies yield satisfactory outcomes when addressing optimization problems characterized by low-dimensional variables and moderate search difficulty, they become substantially less applicable to high-dimensional, complex tasks where the underlying nonlinear fitting relationships remain poorly elucidated. Consequently, there exists an urgent imperative to pursue advanced strategies for enhancing swarm intelligence algorithms, thereby enabling high-precision parameter identification and effectively addressing the critical challenge of improving the accuracy of dynamic gravimetry.

Swarm intelligence optimization algorithms have been widely applied across various fields, including medical resource allocation, investment risk management, path planning, logistics scheduling, and task decision-making, demonstrating considerable potential and value [22,23,24]. Algorithms derived from distinct heuristic inspirations exhibit characteristic advantages, yet their optimization performance varies considerably across different problem types. According to the “No Free Lunch” theorem [25], no single algorithm can achieve uniformly superior performance across all problem domains. Therefore, to address the solution of complex optimization problems, it is essential to transcend the constraints imposed by individual models and to investigate hybrid optimization strategies that offer enhanced generality and robustness.

Prabhat R. Singh et al. proposed the Ludo Game-based Swarm Intelligence (LGSI) algorithm, which simulates the rules of the Ludo board game to select a “winner” from among four constituent algorithms for problem solving [26]. However, this approach is confined to probabilistic selection among only four algorithms, thereby limiting its general applicability. Zhu Haoran similarly invoked the concept of co-evolution, albeit solely for facilitating communication among individuals within a population, rather than addressing co-evolution between distinct algorithms [27]. In 2024, Sharaf Alzoubi et al. introduced the Synergistic Swarm Optimization Algorithm (SSOA), which integrates principles of swarm intelligence and cooperative synergy to enhance exploratory behavior and intensify exploitation of promising solution regions [28]. Nevertheless, these methods remain circumscribed to co-evolution within the population of a single algorithmic framework. Furthermore, hybrid improvement strategies that combine two distinct algorithms have been successively advanced. Examples include the integration of the Bird Swarm Optimization algorithm into the position-updating mechanism of the SSA, as well as combinations such as Ant Colony Optimization (ACO) with Genetic Algorithm (GA), PSO with GA, and PSO with Grey Wolf Optimizer (GWO) [29,30,31]. Contemporary improvement strategies predominantly emphasize rudimentary amalgamation of algorithms, resulting in only marginal enhancements to overall optimization performance. Research concerning the efficient integration of multiple algorithms to further augment optimization accuracy, stability, and universality—thereby realizing genuine inter-algorithm co-evolution—remains notably underdeveloped and constitutes a domain urgently warranting further investigation.

Furthermore, intelligent approaches based on neural networks and machine learning have achieved significant progress in error compensation. Zhou X. et al. embedded the Mind Evolutionary Algorithm and AdaBoost into a BP neural network, using carrier position to estimate gravity disturbances and suppress inertial navigation error propagation, reducing position error by 28% [32]. Gao et al. employed a multi-layer feedforward neural network trained on EIGEN-6C4 model data to estimate gravity vectors, improving radial position accuracy by 31.43% in vehicle tests [33]. Zhang B. Y. proposed an improved fully connected deep neural network for terrain gravity field modeling, achieving mGal-level accuracy with strong generalization across varied terrains [34]. High-performing and well-designed neural architectures serve as a critical driver for breakthrough performance in numerous tasks. However, manual architecture design not only demands substantial human effort, time, and computational resources but also relies heavily on extensive expertise in network training. Even so, there is no guarantee of achieving an optimal model structure, which severely constrains their practical effectiveness and deployment potential.

Neural architecture search (NAS) has emerged as an automated approach for discovering optimal network architectures [35]. It involves defining a suitable search space, employing specific strategies to automatically explore architectural parameters, and iteratively evaluating their performance to identify the best-performing structure for a given task. Commonly adopted and well-established search strategies include reinforcement learning-based, gradient-based, evolutionary algorithm-based, and random search methods [36]. The dynamic gravity measurement data studied in this paper represents typical time-series data. For error compensation based on machine learning, using LSTM—which captures sequential dependencies—is a reasonably sound choice as the core model. However, inherent limitations of recurrent neural networks (RNNs), such as gradient instability, complex cell structures, and substantial computational costs, hinder the effective application of reinforcement learning and gradient-based NAS methods to LSTMs. These feasibility challenges pose significant obstacles for researchers, rendering such strategies unsuitable for automated design of recurrent architectures like LSTM. In contrast, evolutionary algorithm-based strategies are gradient-free optimization methods. A key advantage is their lack of preset search boundaries, enabling exploration in broader search spaces—a characteristic well-aligned with the combinatorial nature of NAS. Extensive applied research has demonstrated their excellent practical performance. Random search, as the name implies, samples architectures randomly from the search space for evaluation. As the simplest and most fundamental strategy, it is often regarded as a low-intelligence algorithm due to its blindness, stochasticity, and instability. Nevertheless, its search performance remains highly competitive and is often hard to surpass, making it commonly used as a baseline algorithm for comparison [37].

Currently, the error mechanisms in platform-based dynamic gravity measurements are not fully understood, and the accuracy, reliability, and generalizability of existing error models remain questionable. Moreover, conventional swarm intelligence algorithms used for parameter identification exhibit notable drawbacks, which severely restrict the practical accuracy and universal applicability of dynamic gravity measurement, particularly in highly dynamic environments. Although machine learning-based error compensation methods can temporarily bypass the need for explicit physical models by training a black box to capture the implicit functional relationships between inputs and outputs, such approaches still lack reliability and stability due to the absence of a solid mathematical foundation. Moreover, the optimal configuration of model architectures and hyperparameters remains a critical challenge that requires urgent resolution.

In response, this paper begins with the fundamental principles of platform-based gravimetry, providing a detailed derivation of the error propagation process and establishing a rigorous theoretical foundation for analyzing the mechanisms of error influence. Based on this analysis, a polynomial error compensation model is constructed. The parameters of this model are accurately identified using a novel swarm intelligence optimization algorithm proposed in this study, which effectively overcomes the notable limitations of conventional optimization methods, thereby enabling reliable compensation for modelable errors in dynamic measurements. Subsequently, a machine learning compensation approach based on LSTM is developed to address non-modelable errors. To optimize the architecture and hyperparameters of the LSTM model, a NAS mechanism driven by the proposed swarm intelligence algorithm is employed. This strategy mitigates the arbitrariness and randomness inherent in manual model design, thus overcoming the performance degradation associated with subjective structural choices. Most importantly, this study innovatively proposes a two-stage error compensation strategy that integrates both modeling approaches. This hybrid framework effectively combines the strengths of the two methods, achieving more stable, reliable, comprehensive, and accurate suppression of dynamic errors. The innovations and contributions of this work are summarized as follows.

1. A Swarm Cooperation Evolution Strategy (SCES) is proposed. Inspired by social evolution, the proposed SCES incorporates five core mechanisms to enable multi-algorithm cooperative optimization, which effectively synthesizes the advantages of individual algorithms. Systematic validation on the CEC2022 benchmark functions confirms its powerful optimization performance, strong generalization ability, and high stability.
2. Polynomial error compensation model guided by mechanism analysis and SCES-driven parameter identification. Based on the principles of dynamic gravity measurement and a systematic error propagation mechanism analysis, a theoretically grounded polynomial compensation model is constructed. The SCES is employed to achieve high-accuracy parameter identification, leading to significant improvement in dynamic gravity measurement accuracy after compensation.
3. Machine learning compensation model optimized via NAS. An LSTM network-based dynamic error compensation framework is developed. Innovatively, NAS technology is leveraged to autonomously determine optimal network architectures and hyperparameter configurations, enhancing modeling accuracy and efficiency.
4. Hybrid strategy with two-stage sequential compensation. A novel strategy creatively integrates the strengths of mechanism modeling and machine learning, which prioritizes systematic error compensation using the polynomial model, followed by residual nonlinear error suppression through the NAS-optimized LSTM model, achieving comprehensive and more precise dynamic error mitigation.

The remainder of the paper is organized as follows. Section 2 details the fundamentals of dynamic gravity measurement and the error influence mechanism analysis, as well as the underlying principle of the adopted LSTM. The inspiration, model, and implementation steps of the proposed SCES are described in detail in Section 3. Section 3 also conducts extensive performance tests and algorithmic comparisons based on the CEC2022 test sets to demonstrate the excellent performance of the proposed algorithm. The proposed complete algorithmic models for dynamic error compensation are presented in Section 4. Section 5 presents a comprehensive experimental validation of the proposed method, whose results and accompanying discussion convincingly demonstrate its feasibility and effectiveness. Finally, Section 6 summarizes and presents a prospect of the whole paper.

## 2. Fundamentals

### 2.1. Basic Principles of Dynamic Gravity Anomaly Measurement

Platform gravimeters rely on high-precision gyroscopes and their servomechanisms to provide an inertially stabilized platform, ensuring precise alignment between the *z*-axis accelerometer and the local gravity vector. According to the formulation of Newton’s second law in non-inertial frames, the specific force measured by the accelerometer represents the vector difference between the vehicle’s kinematic acceleration and gravity acceleration [38]. The fundamental equation for gravity measurement in the vertical direction can be expressed as

(1) $$g=-f_{z}+a_{z}$$

where *g* denotes the gravity measurement value, *f_z_* represents the specific force measurement from the vertical accelerometer, and *a_z_* is the vertical acceleration of the platform. Beyond satisfying Equation (1), dynamic gravity measurements require the application of error correction terms induced by platform motion, followed by subtraction of the normal gravity value from an Earth model to derive gravity anomaly. The mathematical model for gravity anomalies obtained via platform gravimeters is expressed as follows [39].

(2) $$\Delta g_{U}=f_{z}-\gamma -a_{z}+\Delta G_{E}+\Delta G_{H}$$

where *γ* denotes the normal gravity correction, $\Delta G_{E}$ and $\Delta G_{H}$ represent the Eötvös correction and platform tilt correction induced by platform motion, respectively. Their detailed expressions are given by Equations (3)–(5). The normal gravity calculation formula provided in Equation (3) is derived from the World Geodetic System 1984 model, with values solely dependent on platform position [40].

(3) $$\gamma =\frac{9.780325\times \left(1+5.3024\times 10^{-3}\sin^{2}\varphi -5.82\times 10^{-6}\sin^{2}2\varphi \right)}{{\left(1+h/\sqrt{R_{M}R_{N}}\right)}^{2}}$$

where φ denotes the platform latitude, *h* represents the platform height, and *R_M_* and *R_N_* are the Earth’s meridian radius of curvature and prime vertical radius of curvature, respectively.

The Eötvös correction is an essential compensation term accounting for additional centrifugal forces generated by the platform’s motion relative to the rotating Earth. Given the relatively slow velocities of land vehicles on the Earth’s surface, an approximated formula under the ellipsoidal hypothesis suffices for practical requirements [41].

(4) $$\Delta G_{E}=2\omega_{ie}v_{e}\cos\varphi +\frac{v_{e}^{2}}{R_{N}+h}+\frac{v_{n}^{2}}{R_{M}+h}$$

where $\omega_{ie}$ denotes the Earth’s angular velocity, and *v_e_* and *v_n_* represent the platform’s eastward and northward velocity components, respectively. Owing to dynamic operational environments and limitations in stabilized platform control technology, inertial platforms inevitably exhibit tilt deviations. A non-horizontal orientation causes gravimeter outputs to not represent the true vertical acceleration, necessitating essential compensation. According to the principle of rotational invariance, the magnitude of acceleration experienced by any object remains unchanged under orthogonal coordinate system rotation. Given that horizontal disturbance accelerations are significantly smaller than gravitational acceleration *g*, the platform tilt correction is derived as follows [13].

(5) $$\Delta G_{H}=\frac{f_{x}\cdot a_{e}+f_{y}\cdot a_{n}}{g_{m}}$$

where *f_x_* and *f_y_* denote the specific force outputs of the horizontal accelerometers along the *x*- and *y*-axes, *a_e_* and *a_n_* represent the platform’s eastward and northward kinematic accelerations, and *g_m_* is the measured gravity value from the platform gravimeter.

### 2.2. Error Mechanism Analysis of Dynamic Gravity Measurement

By performing variational differentiation calculations on the basic principle Equation (2) of dynamic gravity measurement, the error model is obtained as follows.

(6) $$d\Delta g_{U}=\delta f_{z}-\delta \gamma -\delta a_{z}+d\Delta G_{E}+d\Delta G_{H}$$

where $d\Delta g_{U}$ denotes the gravity anomaly measurement error, $\delta f_{z}$ represents the specific force measurement error, originating from accelerometer bias, scale factor errors, misalignment errors and so on. The normal gravity calculation error δγ depends solely on position inaccuracies. $\delta a_{z}$ represents the vertical acceleration error, resulting from altimetry noise and numerical differentiation errors. In addition, $d\Delta G_{E}$ and $d\Delta G_{H}$ correspond to the Eötvös correction calculation error and platform tilt correction error, respectively, the error analysis of which requires partial differentiation with respect to each variable derived from Equations (4) and (5), satisfying

(7) $$\begin{array}{cc} d\Delta G_{E} & =\frac{\partial \left(\Delta G_{E}\right)}{\partial \varphi }\cdot \delta \varphi +\frac{\partial \left(\Delta G_{E}\right)}{\partial v_{e}}\cdot \delta v_{e}+\frac{\partial \left(\Delta G_{E}\right)}{\partial v_{n}}\cdot \delta v_{n}+\frac{\partial \left(\Delta G_{E}\right)}{\partial R_{N}}\frac{\partial R_{N}}{\partial \varphi }\cdot \delta \varphi \\ & =\left[-2\omega_{ie}v_{e}\sin\varphi -\frac{v_{e}^{2}\cdot R_{N}\cdot k\left(\varphi \right)}{{\left(R_{N}+h\right)}^{2}}\right]\cdot \delta \varphi +\left(2\omega_{ie}\cos\varphi +\frac{2v_{e}}{R_{N}+h}\right)\cdot \delta v_{e}+\frac{2v_{n}}{R_{M}+h}\cdot \delta v_{n} \end{array}$$

(8) $$\begin{array}{cc} d\Delta G_{H} & =\frac{\delta \left(f_{x}\cdot a_{e}+f_{y}\cdot a_{n}\right)\cdot g_{m}-\delta g_{m}\cdot \left(f_{x}\cdot a_{e}+f_{y}\cdot a_{n}\right)}{g_{m}^{2}}\\ & =\frac{1}{g_{m}}\left(a_{e}\delta f_{x}+f_{x}\delta a_{e}+a_{n}\delta f_{y}+f_{y}\delta a_{n}\right)-\frac{\Delta G_{H}}{g_{m}}\delta g_{m} \end{array}$$

where $k\left(\varphi \right)=\left(e^{2}\sin\varphi \cos\varphi \right)/\left(1-e^{2}\sin^{2}\varphi \right)$.

Dynamic gravity measurements typically require the integrated navigation system of global navigation satellite system (GNSS) and inertial navigation system to provide high-precision position, velocity, and platform kinematic acceleration. Therefore, among the dynamic errors induced by the maneuvering characteristics of the vehicle, the measurement errors of the navigation parameters play a relatively minor role. The primary sources of error are the frequently changing velocity, acceleration, and continuously varying position information.

The positioning and velocity measurement errors of the integrated navigation system, as well as the specific force measurement errors of the triaxial accelerometer, are generally small in magnitude and often contain significant high-frequency components. After low-pass filtering, these errors can be approximately treated as constants to simplify calculations. Therefore, the first three terms in Equation (6) can be defined as a constant error *k*_1_. By treating the latitude, velocity, and acceleration of the vehicle as core variables and regarding the products of navigation parameter errors, specific force measurement errors, and other constants in Equations (7) and (8) as constants, the resulting approximate error analytical equation can be simplified as

(9) $$d\Delta {\tilde{g}}_{u}=k_{1}+k_{2}v_{e}\sin\varphi +k_{3}v_{e}^{2}k\left(\varphi \right)+k_{4}\cos\varphi +k_{5}v_{e}+k_{6}v_{n}+k_{7}\frac{a_{e}{+a}_{n}}{g_{m}}+k_{8}\frac{f_{x}{+f}_{y}}{g_{m}}+k_{9}\frac{\Delta G_{H}}{g_{m}}$$

where *k*_1_–*k*_9_ are model coefficients associated with navigation parameter errors, specific force measurement errors, and other constants.

### 2.3. Underlying Principle of the Adopted LSTM

Owing to the inherent complexity and stochasticity of platform maneuvers, conventional error suppression methodologies exhibit constrained efficacy in dynamic gravimetry applications. Leveraging the robust capacity of machine learning for nonlinear fitting and the extraction of latent relational structures, the construction of intelligent compensation models grounded in machine learning constitutes a reliable approach for enhancing measurement precision.

Accurate modeling of dynamic gravimetric errors necessitates the capture of both long-range temporal dependencies and intricate nonlinear sequential characteristics. In contrast to the relatively simplistic architectures of conventional RNNs and Gated Recurrent Units, LSTM networks—through the synergistic interplay of gating mechanisms and the maintenance of an independent cell state—afford more precise regulation of information retention and decay. This architectural design endows LSTM with robust sequential modeling capabilities, thereby effectively mitigating the challenges of vanishing and exploding gradients [42,43]. Furthermore, given the comparatively limited scale of typical gravimetric datasets, LSTM demonstrates superior capacity for capturing temporal dependencies under data-scarcity conditions relative to large-scale models such as Transformers or Mamba architectures. Additionally, LSTM offers distinct advantages in terms of computational tractability and model interpretability, attributes that facilitate subsequent refinement and optimization of the modeling framework [44]. More importantly, empirical evidence indicates that suitably optimized LSTM architectures, when deployed for error compensation tasks, can fully satisfy the operational requirements of gravimetric measurement accuracy. In this context, the judicious selection of a parsimonious yet well-adapted model holds greater practical engineering significance than the indiscriminate pursuit of emerging large-scale paradigms. Accordingly, this study adopts LSTM as the core architectural framework for error compensation. These make LSTM not merely an alternative but a necessary solution for high-precision error compensation. The mathematical formulations for the LSTM are presented below.

The LSTM represents a variant of RNN, which is defined by the following key components: the current cell state ***c****_t_*, the candidate cell state ${\tilde{c}}_{t}$, and the output ***h****_t_*, enhancing traditional RNN architectures through sophisticated gating mechanisms. The cell state ***c****_t_* preserves long-term dependencies within sequential data through three specialized gating mechanisms. The forget gate (***F****_t_*) regulates the retention of information from the prior state ***c****_t_*_−1_. The input gate (***I****_t_*) controls the integration of new memory derived from ${\tilde{c}}_{t}$ into the updated state. Finally, the output gate (***O****_t_*) generates the final hidden state ***h****_t_* by modulating the activated cell state [45].

(10) $$\begin{array}{l} I_{t}=\sigma \left(W_{i}h_{t-1}+U_{i}x_{t}+b_{i}\right)\\ F_{t}=\sigma \left(W_{f}h_{t-1}+U_{f}x_{t}+b_{f}\right)\\ O_{t}=\sigma \left(W_{o}h_{t-1}+U_{o}x_{t}+b_{o}\right)\\ {\tilde{c}}_{t}=\tanh\left(W_{c}h_{t-1}+U_{c}x_{t}+b_{c}\right)\\ c_{t}=F_{t}*c_{t-1}+I_{t}*{\tilde{c}}_{t}\\ h_{t}=O_{t}*\tanh\left(c_{t}\right) \end{array}$$

where ***x****_t_* denotes the input signal, *σ* represents the sigmoid activation function, *tanh* is the hyperbolic tangent activation function, and “*” signifies element-wise multiplication. The weight matrices W* and U* correspond to gate-specific parameters, while b* denotes the associated bias terms.

## 3. The Proposed Swarm Cooperation Evolution Strategy

Accurate identification of the parameters governing the error compensation model is pivotal to the optimal performance of the overall framework. This imperative becomes particularly pronounced when confronted with optimization problems characterized by elevated variable dimensionality, increasingly intricate influence mechanisms, and more opaque nonlinear fitting relationships—conditions that collectively impose substantially heightened demands on the efficacy of parameter optimization methodologies. Consequently, there exists a pressing need to develop an intelligent optimization algorithm that exhibits superior search precision, enhanced robustness, and broad applicability across diverse and complex problem landscapes. This section first presents the inspiration for the proposed SCES, discusses the mathematical model and implementation steps of the algorithm in detail, and concludes with a discussion of optimization performance and computational complexity.

### 3.1. Inspiration

Cooperation evolution refers to a process wherein distinct individuals within a population engage in mutual cooperation and joint evolutionary progression, thereby attaining a more advanced mode of survival and development. In the context of societal co-evolution, this process encompasses intra-group phenomena such as internal information sharing, team reorganization, personnel redeployment, and the dual mechanisms of elimination and recruitment. Simultaneously, it involves inter-group dynamics including information exchange, collaborative teamwork, performance evaluation, and competitive acquisition, as well as macro-level market-driven processes such as the selective allocation of resources and preferential support toward dominant collectives. Inspired by these phenomena, this paper develops a SCES that integrates multiple constituent algorithms and facilitates their efficient synergistic fusion.

**Figure A1:**
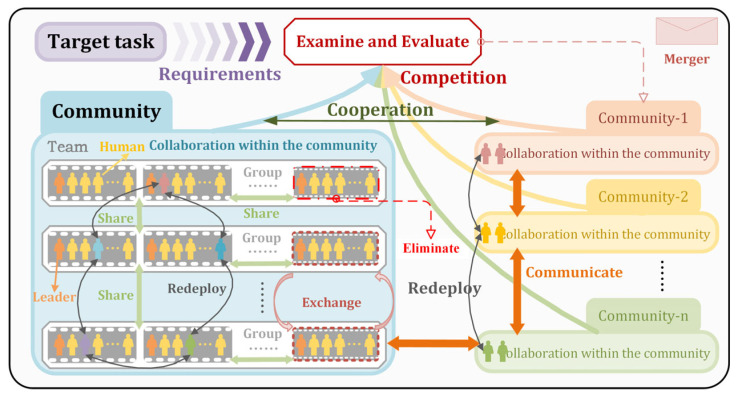
Conceptual diagram of collaborative evolution mechanism.

**Figure A2:**
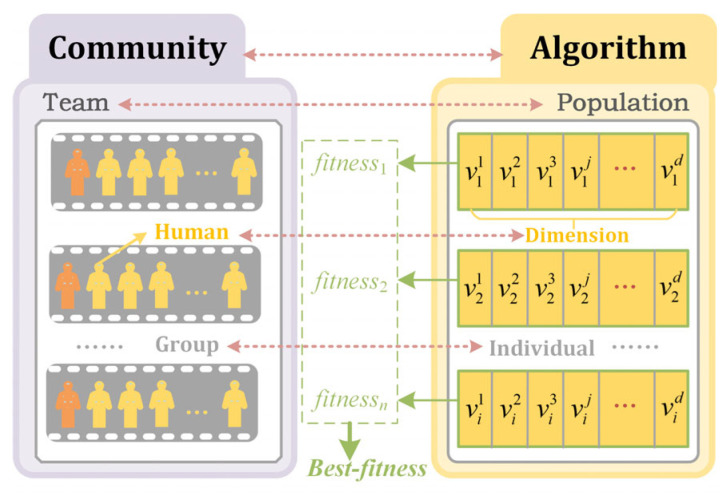
The correspondence between social groups and concepts in algorithmic models.

To concretely illustrate the conceptual underpinnings of swarm co-evolution, a schematic representation is provided in Figure A1 of Appendix B. In this depiction, competitive relationships are first established among distinct collectives. Subsequently, intra-group mechanisms—such as member recruitment, pruning, and redeployment—alongside inter-group synergistic coordination, enable the respective groups to complement one another’s strengths and mutually reinforce their capabilities, thereby enhancing overall task performance. Conversely, collectives ill-adapted to the evolving societal landscape are progressively eliminated or assimilated during the co-evolutionary process. The correspondence between societal entities and the mathematical constructs within the algorithmic model is delineated in Figure A2 of Appendix B, wherein collectives, teams, sub-teams, and individuals are mapped one-to-one onto base algorithms, populations, individuals, and problem dimensions, respectively. The fitness function value quantitatively characterizes the efficacy with which a given sub-team accomplishes the assigned task, and the pursuit of the optimal solution is thus equivalent to identifying the sub-team that exhibits the most exemplary performance within the co-evolutionary framework.

### 3.2. Mathematical Model of Cooperation Evolution

To simplify the description of the mathematical model, the complex process of cooperative evolution is distilled into five core operational mechanisms, including sharing and communication, assessment and evaluation, selection and allocation, elimination and recruitment, and merger and monopoly. The detailed implementation of each mechanism is as follows.

#### 3.2.1. Sharing and Communication

An effective sharing and communication mechanism forms the foundation of cooperative evolution among multiple groups. By engaging in mutual learning and leveraging each other’s strengths to offset weaknesses, each group can evolve to play a more significant role. This collective progress and development not only enhance the individual groups but also drive the evolution of the entire system, ultimately fostering a cohesive community that maximizes overall benefits.

**Figure 2:**
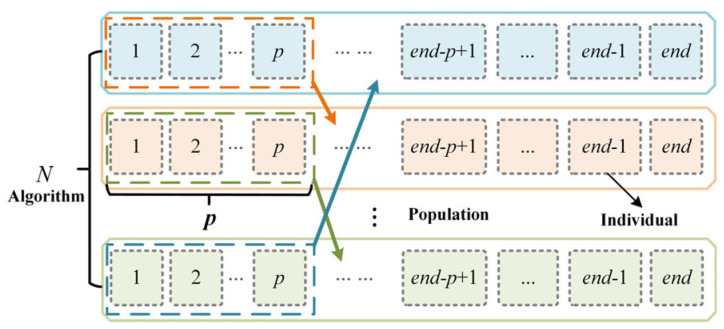
Schematic diagram of the sharing process.

Sharing is achieved by randomly exchanging multiple optimal individuals among the respective algorithms, akin to position redeployments within a company. As illustrated in Figure 2, *N* algorithms each select *p* optimal individuals and randomly reassign them to one of the other algorithms. Simultaneously, each algorithm receives *p* optimal individuals from another algorithm, while discarding its original set of *p* optimal individuals. This redeployment-based sharing mechanism effectively breaks down barriers between algorithms, enabling seamless information exchange. By acquiring advanced individuals from other algorithms and simultaneously discarding their current optimal individuals, algorithms can more easily escape local optima and enhance their exploration capabilities, thereby improving overall algorithmic performance.

Communication refers to the operation process of two individuals generating two new individuals through arithmetic operations, increasing the diversity of population individuals to improve the global search ability of the algorithm and the ability to jump out of local optimum. The communication operation adopts real number coding and arithmetic crossover, and let the two crossover individuals be $s_{1}=\left(v_{1}^{1},v_{1}^{2},\ldots ,v_{1}^{d}\right)$ and $s_{2}=\left(v_{2}^{1},v_{2}^{2},\ldots ,v_{2}^{d}\right)$. Determining whether to implement the crossover operation or not by the crossover probability *P_c_*, and then selecting some variables among the parent individuals to implement the arithmetic crossover by the probability *P_c_* when the crossover is carried out.

Firstly, *d* random real numbers in the interval [0, *P_c_*] are randomly generated and rounded, and then the positions *P*_1_, *P*_2_, …, *P_m_* with the value of 1 are identified from them, i.e., they are the variables for performing crossover, and then *m* random real numbers *a_i_* (*i* = *P*_1_, *P*_2_, …, *P_m_*) in the interval [0, 1] are randomly generated, and the two offspring after the crossover operation can be expressed as

(11) $$\left\{\begin{array}{l} s_{1}^{\prime i}=a_{i}v_{1}^{i}+\left(1-a_{i}\right)v_{2}^{i}\\ s_{2}^{\prime i}=a_{i}v_{2}^{i}+\left(1-a_{i}\right)v_{1}^{i} \end{array}\right.,i=P_{1},P_{2},\ldots ,P_{m}$$

The arithmetic crossover of adaptive crossover is to adaptively vary *P_c_* according to the state of fitness distribution of the population in order to reach a trade-off between exploration and exploitation. It increases *P_c_* when the fitness of individuals in the population converges or tends to be locally optimal and decreases *P_c_* when the population fitness is more dispersed. At the same time, individuals whose fitness is higher than the average fitness of the population correspond to a lower *P_c_*, allowing these individuals to be conserved and enter the next generation, while individuals whose fitness is lower than the average fitness correspond to a higher *P_c_*, allowing this group of individuals to be eliminated. The calculation is shown in Equation (12).

(12) $$P_{c}=\left\{\begin{array}{l} P_{c1}-\frac{\left(P_{c1}-P_{c2}\right)\left(f-f_{max}\right)}{f_{max}-f_{avg}},\;f\geq f_{avg}\\ P_{c1},\;f<f_{avg} \end{array}\right.$$

where *P_c_*_1_ and *P_c_*_2_ are the adaptive upper and lower ranges of the crossover operator, respectively, *f_max_* is the largest fitness value in the population, *f_avg_* is the average fitness value of the population per generation, and *f* is the larger fitness value of the two individuals to be crossed.

#### 3.2.2. Assessment and Evaluation

The fitness value reflects the advantages of individual populations but does not adequately evaluate the overall performance of an algorithm during swarm cooperative evolution. Drawing inspiration from the general principles of social operation, social groups that demonstrate exceptional performance often receive preferential treatment and resource allocation. Similarly, in cooperative evolution, a robust assessment and evaluation mechanism for groups—i.e., the performance evaluation of algorithms—is a critical component. The evolution curve of an algorithm’s fitness captures its historical performance and indicates its future optimization trends, serving as a vital basis for performance evaluation. Building on this concept, we propose the following evaluation criteria to guide the process effectively.

In order to provide a more scientific and comprehensive performance evaluation of the algorithms, we consider several indicators, including the a priori indicator *P_p_*, accuracy indicators *A*_1_ and *A*_2_, the efficiency indicator *E*, the fluctuation indicator *D*, and the comprehensive indicator *S*. Different algorithms have their own unique advantages and applicable types of problems, and the fitness of the problem to be solved and the algorithms selected are quantitatively evaluated by the a priori indicator *P_p_*, and $P_{pn}=c,c\in \left(0,1\right),n\in \left[1,N\right]$. The accuracy indicators can be categorized into absolute and relative accuracy, which describe the maximum effect of fitness enhancement relative to all algorithms and the mean value of enhancement effect relative to the respective algorithms after iterative evolution, respectively, and are computed as shown in Equations (13) and (14). The slope of the evolution curve of the fitness value can characterize the optimization speed of the algorithm, and the average evolution slope within the number of iterations is calculated as a measure of the optimization efficiency of the algorithm, and the larger the value, the higher the optimization efficiency is, the better the optimization performance of the algorithm, which is calculated as shown in Equation (15). The fluctuation performance indicator provided by Equation (16) is used to measure the dynamic dependence of the algorithm on the evolutionary operation, and the smaller the value, the higher the approximation of the algorithm to the optimal solution and the better the optimization performance of the algorithm. The comprehensive performance indicator is shown in Equation (17), which is a comprehensive assessment of the algorithm performance through the area of the region formed by the iteration curve and the coordinate axis, and the smaller the area is, the better the algorithm’s comprehensive optimization performance is.

(13) $$A_{1n}=\max\left(f_{k,1},k\in \left[1,N\right]\right)-f_{n,end}$$

(14) $$A_{2n}=\frac{1}{Iter}\sum_{j=1}^{Iter}\left(f_{n,1}-f_{n,j}\right)$$

(15) $$\left\{\begin{array}{l} K_{n,j}=f_{n,j}-f_{n,j-1},K_{n,1}=f_{n,1}\\ E_{n}=mean\left(K_{n,j}\right) \end{array}\right.$$

(16) $$D_{n}=\frac{1}{Iter}\sqrt{\sum_{j=1}^{Iter}{\left(K_{n,j}-E_{n}\right)}^{2}}$$

(17) $$S_{n}=\sum_{j=1}^{Iter}\left(\frac{f_{n,j}+f_{n,j-1}}{2}\right),f_{n,0}=f_{n,1}$$

where *N* is the number of algorithms, *f*_*i*,*j*_ denotes the value of the fitness function for the *j*th generation of the *i*th algorithm, *Iter* is the number of iterations up to the present time, *K*_*i*,*j*_ denotes the evolutionary slope for the *j*th generation of the *i*th algorithm, and *mean*(·) is the function that calculates the mean.

The metrics provided by Equations (13)–(17) are at different magnitudes, so they need to be normalized, and the normalized means are all 1/*N*. If the normalized metrics are greater than the mean, it indicates that the algorithm is superior compared to other algorithms, and then the evaluation result of the algorithm should be further increased based on the a priori probability, and vice versa, it will cause attenuation to the a priori probability, and the magnitudes of increase or attenuation are closely related to the difference between the respective metrics and the mean values. The performance evaluation of the algorithm using a combination of the five metrics and designed accordingly is shown in Equation (18), and the results are used for the selection and allocation of the population.

(18) $${\mathrm{Evaluat}}_{n}=P_{pn}+\frac{n\left(A_{1n}\right)+n\left(A_{2n}\right)+n\left(E_{n}\right)+n\left(1/D_{n}\right)+n\left(1/S_{n}\right)-c_{1}}{c_{2}}$$

where *n*(·) is the normalized computational function of the vector, *c*_1_ and *c*_2_ are the model coefficients, *c*_1_ is 5/N, and *c*_2_ is used to scale the computed values.

#### 3.2.3. Selection and Allocation

**Figure 3:**
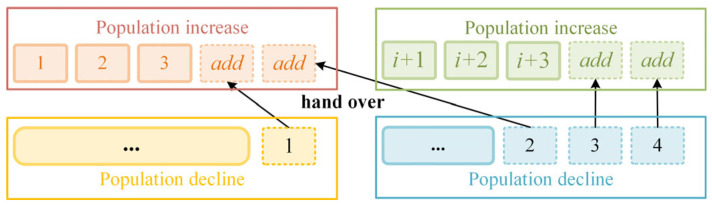
Schematic diagram of the selection and allocation.

The preceding section quantifies the performance of the five base algorithms, with the normalized results serving as a foundation for population reallocation. Algorithms with higher evaluation scores exhibit superior overall performance and, accordingly, should be allocated a larger population size. This approach mirrors real-world dynamics, where teams with greater overall strength are rewarded with increased resource support, fostering further success. The detailed process is illustrated in Figure 3.

The score is defined as the normalized evaluation result of an algorithm, representing the proportion of that algorithm’s strength within the entire algorithm group. In this study, the number of individuals allocated to each algorithm is determined using a roulette wheel approach. The central idea is that algorithms with higher scores occupy larger sectors in the circle, thereby increasing their probability of being selected [46]. To implement this, *M* = 1000 drop trials are conducted. The number of times *m* a drop lands within the area occupied by a specific algorithm is counted, and the number of individuals allocated to that algorithm is calculated as Popsize × *m*/*M*, where Popsize is the total population size. After determining the number of individuals allocated to each algorithm, the allocation method prioritizes maintaining population stability relative to the original algorithm populations. Individuals from algorithms needing a reduction in population are transferred sequentially to algorithms requiring an increase, starting from the algorithms with the lowest demand.

#### 3.2.4. Elimination and Recruitment

Reality can be harsh, as social groups often optimize their members during development. Individuals unable to contribute significantly to the team are inevitably eliminated, with new recruits brought in to fill the gaps. In the proposed SCES, this phenomenon is modeled by removing individuals with poor fitness from the population and introducing new ones. The *P_E_* determines the proportion of individuals to be replaced. The process begins by ranking the population based on fitness values. Individuals corresponding to the *P_E_* ratio in each algorithm are directly eliminated, and an equal number of new individuals are introduced into the population. These new individuals are initialized by perturbing the optimal individuals in the population, ensuring that they are of higher quality than those eliminated.

#### 3.2.5. Merger and Monopoly

In line with the principles of societal operation and the evolutionary law of survival of the fittest, competition among social groups often leads to mechanisms of merger and consolidation. Inspired by this, the proposed SCES incorporates a merger and monopoly mechanism. The timing of mergers, denoted as MergerNum, is predefined, and mergers occur periodically during the iterative evolution process. This mechanism is grounded in the algorithm assessment and evaluation framework described in Section 3.2.2. When the merger timing is reached, the algorithm ranked lowest in the evaluation results is merged. Its population and individuals are transferred to the algorithm ranked highest in the evaluation. Through successive mergers, only one algorithm remains, culminating in a monopoly. Although many algorithms are gradually eliminated during the assessment and evaluation process, their contributions to cooperative evolution are significant.

The five proposed co-evolutionary mechanisms are fundamentally motivated by the objective of emulating, to the fullest extent practicable, the complete co-evolutionary dynamics observed in societal collectives. The omission of any single mechanism would preclude both the logical closure and the functional integrity of the co-evolutionary process. Accordingly, the proposed SCES integrates all five co-evolutionary mechanisms in its design. It is acknowledged that conducting ablation studies on these five mechanisms would permit a direct assessment of the sensitivity of algorithmic performance gains attributable to each constituent component, thereby yielding deeper insights into the functional contributions of individual mechanisms and the underlying rationale for their efficacy. Such an investigation constitutes a valuable direction for future research and warrants dedicated exploration.

### 3.3. Algorithm Architecture and Implementation Process

This section discusses in detail the algorithmic architecture and implementation process of SCES based on the mathematical model described in Section 3.2.

#### 3.3.1. Initialization

The proposed SCES is implemented on the basis of individual algorithms, so firstly the model parameters of the selected base algorithms are initialized. Secondly, according to the optimization problem to be optimized, the dimensions and upper- and lower-bound intervals of feasible solutions are specified, and the basic parameters of the SCES are defined, including the number of populations (Popsize), the number of iterations (IterNum), the timing of cooperation evolution (CENum), and the timing of merger (MergerNum); the values of the crossover probability (*P_c_*), the proportion of elimination (*P_E_*), and the a priori probability (*P_p_*) of the selected base algorithms are assigned an equal probability for the first time.

The population initialization is achieved by randomly generating a number of feasible solutions in the search space. For a *d*-dimensional problem to be optimized with a population size of Popsize, the initialization is shown in Equation (19).

(19) $$v_{i}^{j}{=lb}_{j}+\mathrm{rand}\cdot \left({ub}_{j}-{lb}_{j}\right)$$

where $v_{i}^{j}$ denotes the *j*th dimensional variable of *i*th individual, *i* ∈ [1, Popsize], *j* ∈ [1, *d*], *ub_j_* and *lb_j_* denote the upper and lower bounds of the variable, respectively, and *rand* is a random number between 0 and 1.

#### 3.3.2. Preliminary Optimization Search

Due to the unfamiliarity with the problem type and the performance of the algorithms, before carrying out large-scale iterative optimization, a small number of populations are pre-assigned to each base algorithm, and a number of preliminary iterations are carried out for preliminary optimization. On the one hand, we can quickly understand the optimization performance of the base algorithms for the problem type and also clarify the running time cost of each algorithm under the same number of iterations, so as to lay the foundation for the next in-depth optimization.

First of all, the population initialization is carried out, the same initial population is assigned to each algorithm, and then several iterations are carried out to optimize the algorithms, and the running time of the algorithms is recorded. According to the fitness evolution curve of the preliminary optimization, the operator of assessment and evaluation is invoked to evaluate the performance of the base algorithms, and the results are used as the a priori probability for large-scale iterative optimization on the one hand and used for the selection and allocation of populations on the other hand.

#### 3.3.3. Iterative Optimization

**Figure 4:**
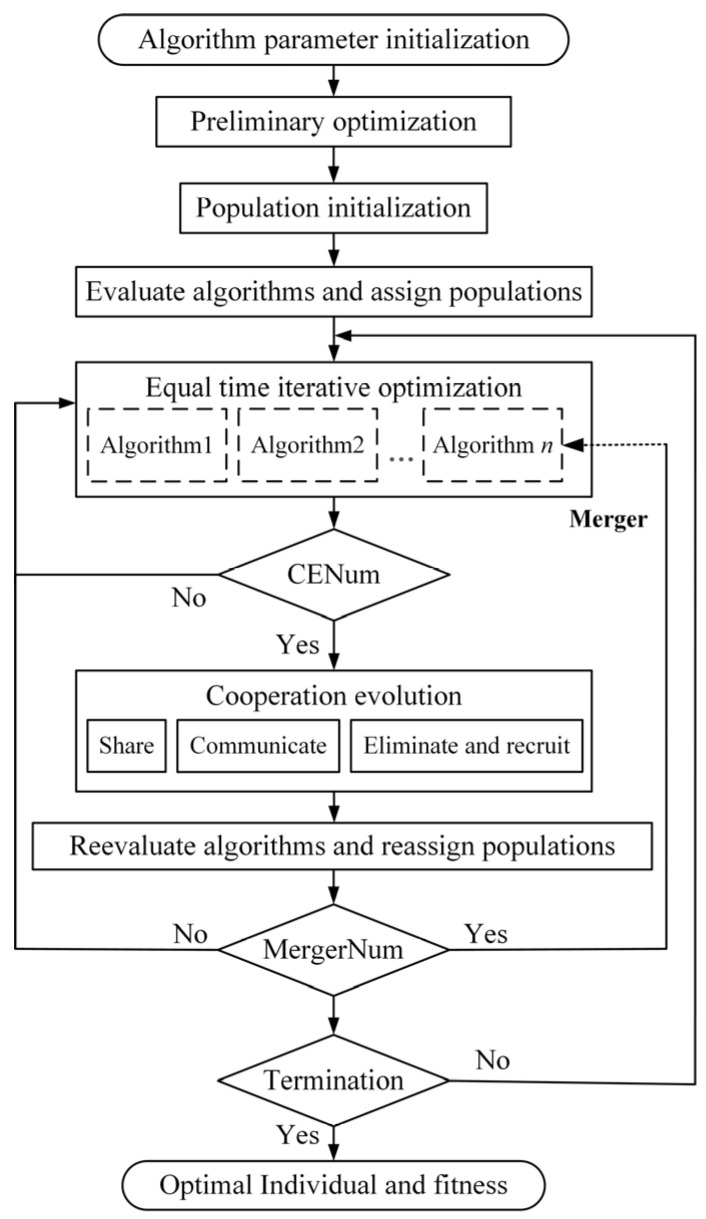
Flowchart of SCES optimization algorithm.

The flowchart of the proposed SCES is given by Figure 4, the dotted lines in the figure indicate that when the merger timing is met, individuals 1 to n of the basic algorithm will be gradually eliminated based on the evaluation results. The implementation steps of iterative optimization search are as follows.

Step 1: Initialization. Initialize a number of populations to Popsize based on the number of preliminary optimization populations. Based on the results of the assessment and evaluation of the algorithms in the initial optimization search, select and assign the respective initial populations for the base algorithms.

Step 2: Iterative optimization search in equal time. Algorithms with lower running time costs can be run more times in the same amount of time. Therefore, iterative optimization is performed by assigning each algorithm the respective corresponding number of iterations according to the inverse of its running time.

Step 3: Cooperation and evolution. When the cooperation evolution timing CENum is reached, the mechanisms of sharing, communication, elimination, and recruitment are executed, and the fitness values of the corresponding individuals are calculated.

Step 4: Selection and allocation. After cooperation and evolution, iterative optimization is carried out, the performance of the algorithms is reevaluated and ranked before the next co-evolution, and the corresponding populations are assigned to each algorithm based on the evaluation results in accordance with the designed selection and allocation mechanism, and then return to Step 2 to continue the iterative optimization.

Step 5: Merger and monopoly. When the merger timing MergerNum is reached, the last one in algorithm evaluation in Step 4 is merged, and the algorithm will no longer be run in the next iteration of optimization. The population individuals belonging to this algorithm will be absorbed by the first one in algorithm evaluation until it reaches the monopoly situation where only one algorithm is left. Otherwise, it returns to Step 2 to continue the iterative optimization.

#### 3.3.4. Analysis of Exploration and Exploitation

Exploration and exploitation are two critical aspects of optimization algorithms, and striking a balance between them is essential for ensuring that the algorithm can accurately and efficiently find the global optimal solution. In the proposed SCES, the iterative optimization of the base algorithms forms the foundation of exploitation, while mechanisms such as sharing, communication, elimination, and recruitment enhance population diversity, thereby strengthening the algorithm’s exploration capabilities. The reallocation of individuals further aids in escaping local optima, improving the algorithm’s ability to explore the solution space effectively. Additionally, the resource-tilting mechanism, driven by selection and elimination, enables superior algorithms to delve deeper into the search space, enhancing their optimization potential. Through these cooperative evolution mechanisms, SCES achieves an efficient integration of multiple algorithms, fostering a balanced development of exploration and exploitation. This balanced approach endows the SCES with significant advantages in terms of optimization accuracy, stability, and overall performance.

#### 3.3.5. Analysis of Computational Cost

The cooperation evolution mechanisms underpinning the proposed SCES predominantly involve addition, logical comparison, and assignment operations, and do not entail iterative looping constructs. Moreover, both the population size and the number of iterations remain consistent with those employed when individual base algorithms are executed independently. Consequently, when addressing optimization problems of moderate difficulty, the computational overhead incurred by SCES is marginally greater than that of a single constituent algorithm, owing to the execution of the co-evolutionary mechanisms. However, given that the total number of iterations required in such scenarios is relatively limited, this increase in computation time is not appreciably significant.

Conversely, when confronted with more challenging optimization landscapes wherein individual base algorithms lack sufficient search capability and are compelled to operate up to their maximum iteration limit, SCES can expediently locate the global optimum and terminate the iterative process prematurely. Under these circumstances, the overall computational burden of SCES may be even lower than that of a single optimization algorithm. In the context of large-scale, high-dimensional, and highly complex problems—where both SCES and individual algorithms must necessarily iterate until the termination criterion is met—the proposed algorithm does indeed incur a tangible drawback in terms of execution time and computational cost. Nevertheless, this trade-off is offset by marked and unequivocal advantages in terms of optimization accuracy, algorithmic stability, and cross-problem generalizability, delivering a level of search efficacy that cannot be attained by any individual base algorithm operating under equivalent computational expenditure. In summary, the enhancement in optimization performance afforded by the proposed algorithm is achieved at the expense of a modest increase in computational burden, which nonetheless remains well within acceptable and practically justifiable bounds.

### 3.4. Performance Tests and Comparisons

In this section, five intelligent optimization algorithms well-established and recognized for their proven performance, namely the Sand Cat Swarm Optimization Algorithm (SCSO) [47], the SSA [48], the Spider Wasp Optimization (SWO) [49], the PSO [50], and the Kepler Optimization Algorithm (KOA) [51], are randomly selected as the basic algorithm individuals (*N* = 5) practicing swarm cooperation evolution. Furthermore, in-depth and extensive performance evaluations were conducted, serving to validate the feasibility of the proposed cooperative evolution mechanisms and provide substantial evidence for assessing their performance.

#### 3.4.1. Test Preparation

Based on the CEC2022 test function set and four different types of typical engineer problems to carry out test experiments to verify the feasibility of the algorithm, independent use of the basic algorithm, the proposed SCES, and the other advanced algorithms optimization test were implemented, and each test function was run independently 100 times. Four quantitative metrics were used to evaluate the effectiveness of the algorithms in various aspects, which are average value (Mean), optimal value (Best), standard deviation (Std) and Rank based on the comprehensive performance in 100 tests. This constitutes a standard methodology within the domain of swarm optimization algorithm performance evaluation, one that has garnered widespread acceptance and extensive application in the literature [52,53]. Furthermore, a Wilcoxon rank-sum test was conducted to compare the proposed algorithm against each respective benchmark algorithm [54]. This statistical analysis serves to ascertain whether significant differences exist between algorithmic pairs, thereby enabling a comprehensive and rigorous assessment of overall algorithmic performance.

The test experiments first determined the total number of iterations (IterNum = 500), pre-allocated 10 populations for each algorithm for 20 iterations during preliminary optimization, and expanded the population size to Popsize = 100 before the start of the formal iterative optimization process. Defined MergerNum = 100, which allows for the periodic and uniform merger of algorithms during the iterative optimization process, and empirically set CENum = 50, *P_c_* = 0.2, and *P_E_* = 0.2. The model parameters of the basic algorithm individuals were set according to the author’s recommendations in the corresponding references. The population size and iterations are kept the same as SCES.

#### 3.4.2. Comparative Validation with Cooperation Fusion-Related Algorithms

The cooperation fusion of multiple algorithms to further improve the accuracy and stability of the optimization solution is the development trend of intelligent optimization algorithms. In this section, SCES is compared with the existing optimization algorithms with multi-algorithm cooperation fusion to fully demonstrate the effectiveness and advancement of the cooperation evolution mechanism proposed in this paper that can efficiently fuse multiple optimization algorithms. The selected comparison algorithms include Ludo Game Swarm Intelligence (LGSI) [26], SSOA [28], Co-evolution Spider Monkey Optimization (CSMO) [27], GA-ACO [29], and PSO-GWO [31], among which LGSI is one of the few examples of multi-algorithm joint use, which initially realizes the synergistic fusion between algorithms. SSOA and CSMO achieved the cooperation evolution of population individuals within the algorithm, implementing a more diverse and comprehensive exploitation of the search space. GA-ACO and PSO-GWO are typical examples of hybrid improvement strategies using two algorithms, which have been proved to have better performance than individual algorithms in real problems.

**Figure A3:**
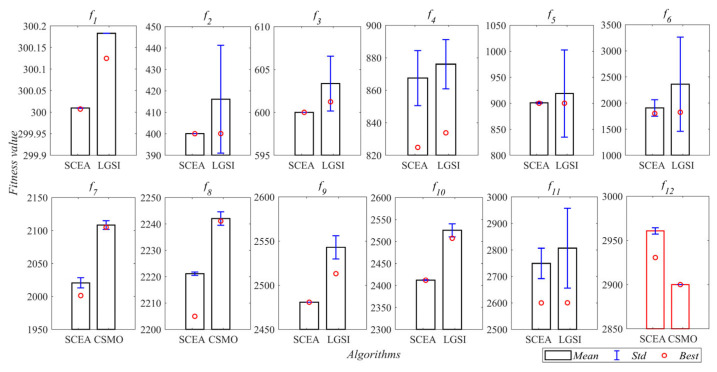
Comparison between SCES and optimal cooperation fusion-related algorithms.

The test sets used in the experiments are derived from the open source and commonly used CEC2022. The CEC2022 consists of 12 test functions of varying difficulty and variety, which are defined as *f*_1_–*f*_12_ and provide significant and varied search obstacles for algorithm testing. For detailed information, please refer to Appendix A. The results of the test experiments are shown in Table 1, and the optimization results of SCES and the optimal comparison algorithms have been bolded and plotted in Figure A3 of Appendix B to visualize the improvement. The bar chart indicating that SCES failed to achieve the best optimization results is highlighted in red in the Figure A3 of Appendix B.

Overall, SCES achieves the best optimization results in 11 of the 12 test functions of CEC2022 except *f*_12_, and the proposed cooperation evolution mechanism has an effectiveness rate of 91.67% compared to various existing cooperation fusion strategies. The average improvement in SCES over the optimal cooperation fusion-related algorithms in the Mean and Best metrics are 3.70% and 1.29%, respectively, of which the Mean of *f*_6_ is most significantly improved by 19.23% compared to the optimal cooperation fusion-related algorithm LGSI. The smallest improvement is the function *f*_1_, which is because the cooperation fusion-related algorithm has achieved the result that is very close to the global optimum on the simpler test function, and the SCES has less room for improvement, and the Mean and Best still improved by 0.0578% and 0.0392% compared with the optimal LGSI. The fact that the test function *f*_12_ does not achieve optimal optimization results is due to the fact that SCES is limited by the optimization performance of the basic algorithm individuals. After analysis, the performance of the five basic algorithms of SCES on this function is not ideal, and their respective search strategies cannot effectively solve this complex and multimodal testing function. Due to the fact that the performance of SCES is based on the underlying algorithm, the performance of SCES will inevitably be affected when all individual underlying algorithms are no longer applicable. Nevertheless, SCES has achieved significant improvements in performance compared to individual base algorithms, demonstrating the effectiveness of the proposed fusion strategy.

LGSI can achieve suboptimal test results in multiple test functions, and the mean value of the comprehensive ranking of the optimization effect is 2.25, which is only second to 1.08 of SCES, while SSOA, GA-PSO, and other algorithms implementing simple fusion mechanisms do not achieve satisfactory optimization results. The results show that LGSI, which probabilistically selects multiple basic algorithm individuals, performs better than other simple fusion algorithms but still has a disadvantage compared to SCES, which has a more in-depth and comprehensive cooperation evolution mechanism.

Table 2 presents the *p* values obtained from the Wilcoxon rank-sum test comparing the proposed SCES against cooperative fusion-related algorithms across all benchmark functions within the CEC2022 test suite. A *p* value below the significance threshold of 0.05 indicates rejection of the null hypothesis, which posits the absence of a statistically significant difference between the two comparators. Values in Table 2 that satisfy *p* < 0.05 are highlighted in bold typeface. From these results, it can be concluded that the outcomes produced by SCES are significantly different from those of the comparison algorithms on the vast majority of the test functions under consideration.

Overall effectiveness (OE) and best reachability (BR) are used to judge the strengths and weaknesses of the algorithms from the perspective of the whole test set, and Equations (20) and (21) give the specific formulas of the two metrics, which are used to characterize the average error of the results obtained by the test algorithm from the optimum and the average probability of achieving the best optimization results among all comparison algorithms [55,56]. The smaller OE is, the closer the algorithm’s average optimization result is to the global optimal solution for all functions in the test set, while the larger BR is, the higher the probability that the algorithm achieves the optimal optimization result for all comparison algorithms, both of which are important metrics of the algorithm’s optimization accuracy.

(20) $$OE=\frac{1}{n}\sum_{i=1}^{n}\left|\frac{F_{i}-F_{b}}{F_{b}}\right|$$

(21) $$BR=\frac{1}{n}\sum_{i=1}^{n}\frac{m-r_{n}}{m-1}$$

where *F_b_* is the theoretical global optimum of the *i*th test function, *F_i_* is the average of the test algorithm’s optimization results for that test function, *n* is the total number of functions in the test function set, *m* is the number of algorithms for comparison, and *r_n_* is the comprehensive performance ranking value of the algorithm in the *n*th test function.

**Figure A4:**
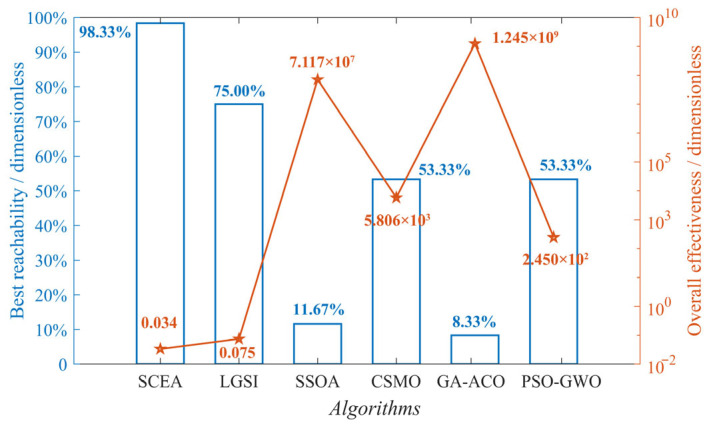
OE and BR of the cooperation fusion-related algorithm on CEC2022.

The OE and BR of the cooperation fusion-related algorithms on the CEC2022 test set are shown in Figure A4 of Appendix B, and the results show that the optimization average value of SCES on CEC2022 is the closest to the theoretical global optimum, with an OE of only 0.034, followed by LGSI with an OE of 0.075. The improvement of OE compared to that of LGSI is 54.67%, and the BR of SCES is as high as 98.33%, which is significantly better than that of the second-place LGSI with 75.0%, and it has a more significant optimization effect compared with the other cooperation fusion-related algorithms.

GA-ACO and PSO-GWO, which simply fused two optimization algorithms, and SSOA and CSMO, which implemented cooperation evolution of population individuals within a single algorithm, did not achieve satisfactory optimization results, which shows that these two fusion improvement strategies did not improve the performance of the optimization algorithms obviously. The performance advantage of LGSI, which selects the optimal choices among multiple optimization algorithms, indicates that the combined use of multiple algorithms is indeed fruitful. More importantly, the SCES proposed in this paper not only can use more basic algorithm individuals but also strengthens the cooperation evolution among them, so it achieves more outstanding optimization performance than LGSI and has significant advantages in terms of optimization accuracy, stability, and generalizability to various problems.

#### 3.4.3. Comparative Test with Single Algorithm Used Independently

Single algorithms used independently are more intensively researched and systematically matured and are the current mainstream techniques for metaheuristic optimization algorithms. In order to fully examine the outstanding performance advantages of SCES over single optimization algorithms, in addition to the selected basic algorithm individuals, this section supplements with four typical optimization algorithms (GWO [57], Whale Optimization Algorithm (WOA) [53], Nutcracker optimization algorithm (NOA) [55], and GA [58]) and three newly proposed metaheuristic algorithms (FATA [59], Lung Performance Optimizer (LPO) [60], Bitterling Fish Optimization (BFO) [61]), to which a fuller comparison test is performed. GWO and WOA are very typical optimization algorithms with strong adaptability and robustness, which are widely used in many engineering optimization problems and show their excellent performance of fast convergence. NOA is a swarm intelligence optimization algorithm proposed in 2022, which has been proved to be suitable for optimization solutions of many complex problems and engineering applications, with high optimization accuracy. GA is the most classical optimization algorithm based on biological evolution, with strong fault tolerance, parallel computing ability, and global search ability, especially suitable for nonlinear, nonconvex, multimodal, and other complex function optimization problems. FATA, LPO, and BFO are the representatives of the latest ones proposed as a way of reflecting the novelty and sophistication of the proposed SCES.

**Figure A5:**
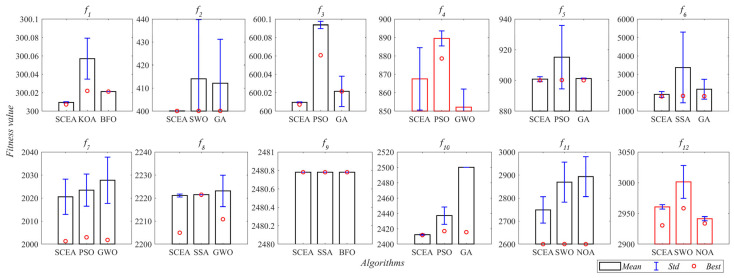
Comparison of test results between SCES, the optimal basic algorithm individual and comparison algorithm.

The evaluations presented in this section are conducted on the same set of 12 CEC2022 benchmark functions shared with Section 4.2. The experimental results are shown in Table 3, and Figure A5 of Appendix B gives a plot of the test results of SCES, the optimal basic algorithm individuals, and the optimal comparison algorithm, the results of which have been bolded in Table 3. The bar chart indicating that SCES failed to achieve the best optimization results is highlighted in red in the Figure A5 of Appendix B.

The experimental results demonstrate that the proposed SCES achieved the best optimization performance in 10 out of the 12 test functions, corresponding to an effectiveness rate of 83.33%. Compared to the best-performing basic algorithm individual, the SCES improved the average and best optimization results by 7.62% and 0.83%, respectively. The most significant improvement in the average value was observed for function *f*_6_, with a 76.81% enhancement over the SSA, while the largest improvement in the best value was recorded for function *f*_4_, exceeding PSO by 6.51%. When compared to the best results obtained by all comparison algorithms, the SCES also achieved average improvements of 2.07% in the mean value and 0.22% in the best value. Notably, the average value for *f*_6_ was improved by 14.92% compared to GA, and the best value for *f*_4_ was enhanced by 1.48% relative to GWO.

However, the improvements for functions *f*_1_, *f*_3_, and *f*_8_ were marginal, which reduced the overall average improvement. This can be attributed to the fact that these functions possess relatively simple landscapes with few obstacles to the global optimum, allowing individual algorithms to achieve high accuracy with limited room for further refinement near the global solution. Nevertheless, the fact that SCES still achieved minor improvements under such conditions demonstrates its consistent ability to escape local optima and enhance search accuracy across diverse function types, highlighting its robust exploration capability. In terms of comprehensive performance ranking, the SCES achieved the best average rank of 1.5, followed by GA (4.33) and NOA (5.67). These results indicate that the SCES consistently maintains top performance, whereas other algorithms exhibit significant performance fluctuations across different test functions due to their inherent characteristics and limitations when used in isolation. This strongly validates that the SCES effectively integrates the strengths of individual algorithms, significantly enhancing universality and stability.

The optimization performance of SCES is closely related to the performance of its constituent algorithms. This explains why SCES did not achieve leading results on functions *f*_4_ and *f*_12_. Analysis reveals that five baseline algorithms—SCSO, SSA, SWO, PSO, and KOA—performed poorly on these functions, all ranking near the bottom. Although SCES did not outperform all advanced algorithms in accuracy on these functions, it still achieved noticeable improvements over its baseline components. For function *f*_9_, SCES did not yield significant improvement due to the simplicity of the function, which leaves little room for enhancement, and the inherent randomness in the optimization process leading to minor variations among algorithms. This does not imply that SCES is inferior to individual algorithms in such cases.

Table 4 reports the *p* values returned by the Wilcoxon rank-sum test, wherein the results obtained from each comparison algorithm are evaluated against the SCES results for every individual test function. Values in Table 2 that satisfy *p* < 0.05 are highlighted in bold typeface. The *p* values presented in the table indicate that, for the vast majority of the benchmark functions, the outcomes yielded by SCES exhibit statistically significant differences relative to those produced by the compared optimization algorithms.

**Figure A6:**
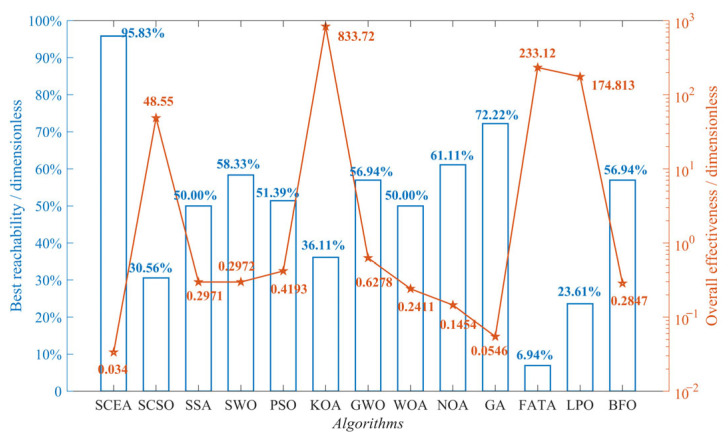
OE and BR of the single algorithm used independently on CEC2022.

Figure A6 of Appendix B illustrates the OE and BR of SCES and individual algorithms on the CEC2022 test set. The results show that SCES achieved the closest average optimization result to the theoretical global optimum, with an OE of only 0.034, followed by GA with an OE of 0.0546. This represents a 37.73% improvement in OE over GA. Furthermore, SCES attained a best achievable rate of 95.83%, significantly outperforming GA (72.22%) and other cooperative or fusion-based algorithms. Compared to the best individual baseline algorithm, SCES improved the BR from 58.33% to 95.83% and reduced the OE from 833.72 to 0.034. It also exhibited a substantial advantage over the best reference algorithm, GA, which achieved a BR of 72.22% and an OE of 0.055. It is worth noting that these results were obtained under the configuration of Popsize = 100 and IterNum = 500. Further improvements in optimization accuracy can be expected with increased population sizes and iteration counts, albeit at higher computational cost.

In summary, SCES demonstrated consistent performance advantages across different test functions. Variations in improvement levels are attributable to the distinct characteristics of each function. Whether for simple functions already near the global optimum or complex functions that are extremely difficult to optimize, the observed improvements are statistically and practically meaningful, strongly validating the effectiveness and advanced nature of the proposed SCES.

## 4. The Complete Algorithmic Models for Dynamic Error Compensation

### 4.1. The Polynomial Error Compensation Method

Based on the error analysis of platform-based dynamic gravity measurement established by Equation (9), a polynomial-based error compensation method is constructed as shown in Equation (22), with this approach designated as Method I.

(22) $$\delta g_{\mathrm{out}}=\delta g_{0}-d\Delta {\tilde{g}}_{U}$$

where $d\Delta {\tilde{g}}_{U}$ is dynamic gravity measurement error, coefficients *k*_1_ to *k*_9_ represent the calibrated parameters of the error compensation model, the term *δg*_0_ denotes the original gravity measurement result, and *δg_out_* represents the gravity output after dynamic error compensation.

The accuracy of model parameters is critical for effective error compensation. This work defines the root mean squared error (RMSE) between the dynamically compensated output and the gravity reference value as the fitness function value. The proposed novel SCES framework is employed for precise parameter identification, thereby elevating the implementation effect of the proposed polynomial error compensation model.

### 4.2. Compensation Method Based on Optimized LSTM via NAS

While polynomial-based error compensation models possess rigorous mathematical foundations, the complex and dynamic environments encountered during gravity measurements introduce highly intricate errors. Beyond cognitive systematic errors, significant residual errors persist, imposing inherent limitations on polynomial compensation efficacy. Machine learning algorithms, particularly LSTM networks, offer substantial advantages in data prediction and fitting by extracting underlying patterns from training datasets [62]. This section establishes a dynamic error compensation model based on optimized LSTM via NAS to achieve deeper error correction of gravity measurements, designated as Method II.

Beyond the inputs utilized in the polynomial model (i.e., *f_x_*, *f_y_*, *g_m_*, *v_e_*, *v_n_*, *φ*, *a_e_*, and *a_n_*), dynamic measurement errors exhibit direct dependence on the carrier’s kinematic state. Consequently, the carrier’s attitude angles (*att_e_*, *att_n_*, and *att_u_*), vertical velocity (*v_u_*) and acceleration components (*a_u_*), and positional coordinates within the navigation frame (longitude *λ* and height *h*) are incorporated as additional inputs to the LSTM model, resulting in 15 input nodes. Following acquisition of the gravity reference values along the survey line, the measurement error, defined as the difference between measured and reference values, serves as the single output node. Experimental data formatted accordingly constitute the sample set, partitioned into training and testing subsets in a 5:1 ratio for model development and evaluation.

High-performing, rationally designed neural architectures serve as engines for performance leaps in numerous tasks. However, manual network design demands substantial time, computational resources, and specialized expertise, yet may still fail to yield optimal architectures, thereby constraining the practical efficacy of dynamic error compensation models. To address this, we implement an evolutionary architecture search for LSTM optimization using the second SCES.

#### 4.2.1. Network Search Space

The optimal LSTM model encompasses both architectural components and hyperparameters. This section defines a comprehensive search space by specifying all optimizable variables. For network architecture, the primary parameters are the number of layers and nodes per layer. Secondary parameters include (1) whether an activation function follows each LSTM layer and its type, enhancing model expressivity through nonlinear transformations, and (2) whether a dropout layer is incorporated and its discard rate, which improves generalization by randomly omitting neurons during training to mitigate overfitting. Collectively, six architectural variables require optimization. Regarding hyperparameters, the model optimizer constitutes the principal tunable element. Additional hyperparameters include initial learning rate, learning rate decay period, learning rate decay factor, batch size, and maximum epoch, yielding six optimizable hyperparameters.

Thus, the search space comprises twelve heterogeneous variables, encompassing discrete and continuous types, including integer and floating-point values, resulting in a substantial and intricate optimization landscape. A hybrid encoding strategy designed to address this complexity is detailed in the following section.

#### 4.2.2. Hybrid Encoding Strategy and Constraint Mechanism

**Figure 5:**
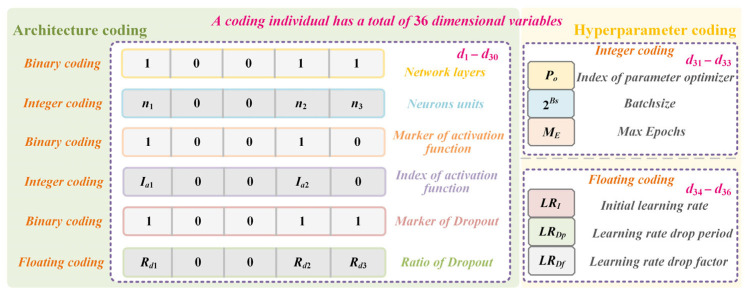
Schematic diagram of search space and hybrid encoding strategy.

A hybrid encoding strategy incorporating integer, floating-point, and binary representations was designed according to variable types and format requirements. Each encoded solution constitutes an individual within the second SCES for iterative optimization. Figure 5 illustrates the search space and hybrid encoding framework, with the specific encoding scheme detailed as follows.

Discrete architectural encoding for the LSTM network employs integer and binary representations. First, the number of layers is encoded in binary, with a maximum layer limit of 5. A value of 1 indicates the actual presence of an LSTM layer, whereas layers encoded as 0 are omitted without parameter retention. Subsequently, neuron counts per existing LSTM layer are encoded as integers within [8, 128]. Next, activation function flags are generated with length corresponding to the actual layer count. These binary flags determine activation function presence. A value of 1 signifies an activation layer immediately following the LSTM layer; otherwise, no activation is applied. The activation type index is integer-encoded within [1, 4], denoting sigmoid, tanh, ReLU, and linear functions, respectively. This index remains valid only when the activation flag equals 1. Dropout configuration follows an analogous scheme. Dropout flags (binary-encoded with layer-determined length) indicate dropout layer presence, where 1 triggers insertion after the LSTM layer, and 0 omits it. The dropout ratio index *i* is integer-encoded within [0, 9]. During decoding, this index maps to the discard rate via 0.05 + 0.05 × *i*, yielding values in [0.05, 0.5]. This index is active only if the corresponding dropout flag is 1.

Hyperparameter encoding employs distinct strategies per parameter type. Optimizer selection utilizes integer categorization. A randomly generated floating-point number within [1,5] is rounded up via the ceiling function to yield a categorical index mapping to ‘*adam*’, ‘*sgdm*’, ‘*rmsprop*’, ‘*adagrad*’, or ‘*nadam*’. Initial learning rates are logarithmically encoded by uniform sampling in log_10_ space. The encoded logarithmic index is a random value ∈ [0, 1], decoding to learning rates spanning [10^−5^, 10^−2^]. Both the learning rate decay period and the decay factor adopt floating-point encoding with random values ∈ [0, 1]. The decoded decay period represents the relative epoch position (proportion of total epochs) where decay initiates. The decay factor is linearly scaled to [0.2, 0.5] during decoding. Batch size implements exponent encoding. Integer exponent *n* ∈ [4, 8] is stored, with the decoded value given by BatchSize = 2*^n^*. Maximum epoch count encodes a random floating-point number ∈ [0, 10], which is rounded up to an integer index. The final epoch count is obtained by multiplying this index by 50.

During population initialization and iterative optimization, constraint conditions are enforced to validate encoded individuals and prevent invalid network architectures. These constraints comprise the following points: (1) dropout layers are excluded from the final network layer; (2) at least one LSTM layer and one activation layer must be present; (3) the final activation layer cannot utilize linear activation (preventing unbounded outputs); (4) for multi-layer LSTM architectures, neuron counts per layer must follow a monotonically decreasing sequence. Guided by this encoding scheme, population initialization and evolutionary optimization proceed via the second SCES.

#### 4.2.3. Fitness Function for NAS

The fitness function serves as the metric for evaluating candidate architectures within the search space, playing a pivotal role in discovering the globally optimal network configuration. Although training-based performance evaluation incurs significant computational costs, its high accuracy provides direct feedback on search efficacy. After training candidate models on the training set, validation set performance is assessed on the resulting architectures. The RMSE between model outputs and ground-truth values constitutes the fitness evaluation metric, where lower fitness values correspond to superior network architectures.

#### 4.2.4. Neural Architecture Search Process

The second SECS used for NAS is defined as Algorithm 1, and its pseudocode for the implementation is presented below. The second SCES is initialized by defining the population size and the number of iterations. Subsequently, an initial population is randomly generated within the network search space according to a hybrid encoding strategy. The positions of individuals within this population are then updated based on the SCES. Following this, the fitness value of the best individual in the current generation is evaluated. If this current fitness value surpasses the historical best fitness value, the optimal individual position is updated; otherwise, the best position from the previous iteration is retained. Upon reaching the maximum number of iterations, the final population is output. The optimal network architecture and hyperparameter settings are then decoded from the best individual according to the hybrid encoding rules.
**Algorithm 1:** The second SCES for NAS1: **Initialization:**
2:    Initialize algorithmic parameters: *Popsize*, *IterNum*, *CENum*, *MergerNum*, *P_c_*, *P_E_*, *P_p_*3:    Initialize population according to random strategy, Equation (19)4:    Conduct preliminary optimization search at a small number of populations and iterations5:    Evaluate base algorithms and assign populations based on preliminary optimization, Equations (12)–(17)6: **while** (*t* ≤ *IterNum*) **do**7:    Iterative optimization search in equal time according to each algorithm’s strategy8:    **if** (*t* mod CENum = 0) **then**9:    Sharing and communication are conducted, Equations (11) and (12)10:     Elimination and recruitment are executed, Section 3.2.411:     Reevaluate algorithms and reassign populations, Equations (12)–(17) and Section 3.2.312:     **if** (t mod MergerNum = 0) **then**13:       The last one in algorithm evaluation is merged, Section 3.2.514:     **end if**15:    **end if**16:    Update the best solution17: **end while**18:    Decoding the best solution**return** optimal architecture and hyperparameters

### 4.3. Two-Stage Hybrid Compensation Strategy

**Figure 6:**
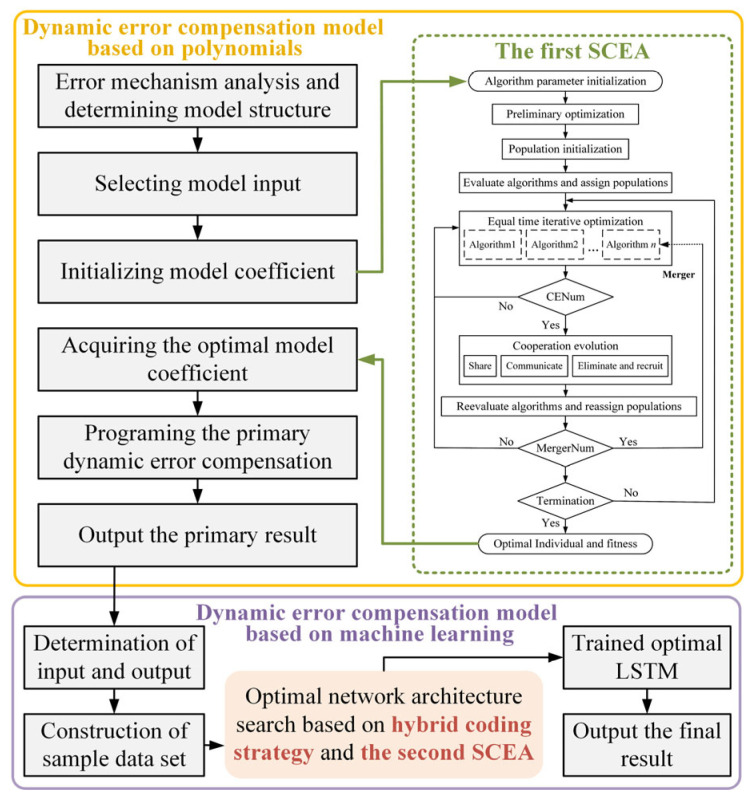
Flowchart of two-stage dynamic error compensation method.

While polynomial-based error compensation effectively suppresses systematic errors during dynamic measurements, residual errors persist and significantly degrade accuracy. To address this limitation, we innovatively propose a hybrid compensation framework integrating both approaches, illustrated in Figure 6, and implement an optimized LSTM model—identified via NAS—to perform deep residual error correction, further enhancing dynamic gravity measurement precision.

The two-stage methodology sequentially applies polynomial fitting followed by optimized LSTM-based refinement, thereby synergizing the advantages of both techniques. This integrated solution not only improves compensation accuracy but also enhances model interpretability, ultimately achieving superior suppression of motion-induced measurement errors during carrier maneuvers. We designate this approach as Method III. For its second-stage LSTM-based error compensation model, the input remains consistent with Method II. However, the model output is modified to the residual error obtained by differencing the preliminary result from the first-stage compensation and the gravity reference value. Crucially, the network architecture requires re-optimization via NAS to obtain the optimal model specifically tailored for residual estimation.

Given that the two compensation methodologies—one predicated on polynomial fitting and the other on machine learning—each harbor inherent limitations while simultaneously exhibiting complementary characteristics, a two-stage compensation strategy that synergistically integrates both approaches is proposed. Specifically, the polynomial fitting-based method is employed to compensate for cognizable, modelable error components, thereby enhancing the reliability and stability of the compensation process. Subsequently, machine learning is leveraged to compensate for the residual, difficult-to-model error constituents, further augmenting the overall accuracy of error mitigation. The proposed framework thus amalgamates the respective strengths of both models while reciprocally redressing their individual deficiencies, and this integration constitutes the principal innovation of the present study. In recognition of the necessity for precise parameter identification within the polynomial error compensation model, and acknowledging that the architectural configuration of the LSTM network directly governs the efficacy of the machine learning-based compensation component, the SCES and a NAS optimization strategy are respectively introduced. The former is dedicated to optimizing the parameters of the polynomial model, whereas the latter is employed to refine the LSTM architecture. Both technical elements are devised to substantiate and enhance the performance of the overarching two-stage error compensation methodology. Consequently, the hybrid two-stage compensation strategy represents the core contribution of this work, with the SCES and NAS-optimized model architecture functioning as enabling mechanisms in service of this central advancement.

## 5. Experiments and Discussion

Acquiring real experimental data for parameter identification and model training of the error compensation model is an essential prerequisite for evaluating the feasibility and effectiveness of the proposed method. Furthermore, compensation experiments under three different maneuvering conditions were designed to validate the practical performance and generalization capability of the approach. This section begins with a detailed description of the experimental setup and data acquisition process. It then elaborates on the procedures and specifics of the experimental validation phase, along with the parameter configurations of the proposed algorithm. Finally, the error compensation results are presented and subjected to in-depth discussion and comprehensive analysis.

### 5.1. Experimental Data Acquirement

#### 5.1.1. Experimental Equipment

**Figure A7:**
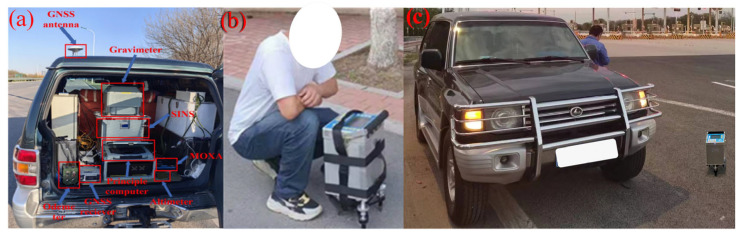
Experimental equipment.

As illustrated in Figure A7 of Appendix B, the experimental vehicle was equipped with a platform-based relative gravimeter independently developed by CSIC 707 Research Institute, a dual-axis laser strapdown inertial navigation system (SINS) manufactured by Xi’an Chenxi Aviation Technology Co., Ltd. in Xi’an, China, a GNSS receiver manufactured by Shanghai Huace Navigation Technology Co., Ltd. in Shanghai, China, a data acquisition computer with ancillary systems manufactured by Advantech Intelligent Technology Co., Ltd. in Kunshan, China, and supplementary navigation sensors such as an odometer (OD) manufactured by Changchun Yuheng Optics Co., Ltd. in Changchun, China and a barometric altimeter manufactured by Xi’an Precision Measurement and Control Co., Ltd. in Xi’an, China. The high-precision navigation data (attitude, velocity, and position) during experiments were provided by a tightly coupled SINS/GNSS/OD/barometer-integrated navigation system, with carrier acceleration derived through first-order differentiation of velocity. The platform gravimeter facilitates dynamic gravity anomaly acquisition, and *f_x_* and *f_y_* are provided by the horizontal accelerometer of the gravimeter. Prior to dynamic measurements, gravity reference values were statically acquired at 2 km intervals along survey lines using a CG-5 high-precision relative gravimeter manufactured by Scintrex (Concord, ON, Canada). These values were interpolated to establish continuous gravity references for dynamic gravity measurements. The key specifications of the instrument are detailed in Table 5.

#### 5.1.2. Experimental Procedure

**Figure A8:**
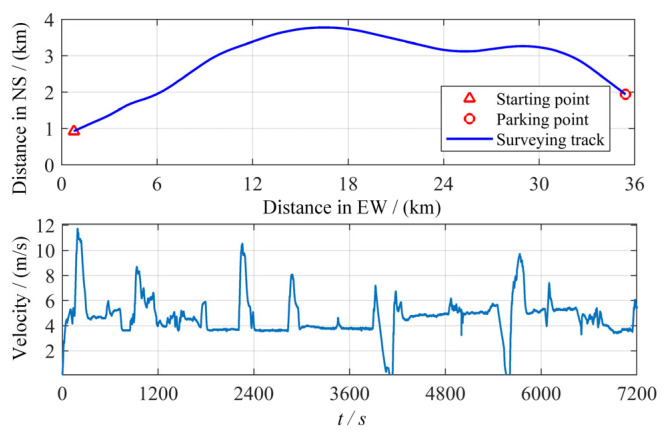
Route and velocity variation in pre-experimental data collection.

**Figure A9:**
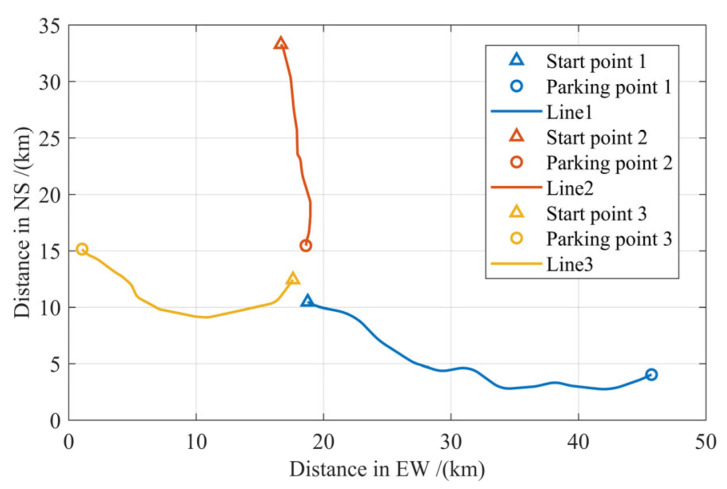
Trajectory of the experiments.

Prior to formal dynamic gravity measurements and error compensation, a 2 h preliminary experiment under 1 Hz sampling protocol was conducted to acquire sufficient data for parameter identification of the polynomial error compensation model and model training of the machine learning method. The resulting model was subsequently deployed for dynamic error compensation during formal dynamic gravity measurement experiments. The experimental process is conducted on a closed road, where the road condition and traffic flow meet the conditions of a Class III road in China’s road classification. The dynamic measurement experiment necessitates the selection of a route whose road conditions, traffic volume, topographical features, and trajectory characteristics collectively satisfy the stringent requirements of gravimetric surveying. The actual trajectory of the selected route is depicted in Figure A8 of Appendix B. As depicted in Figure A9 of Appendix B, the experiment spans approximately 40 km at a mean velocity of 4 m/s, and the route and velocity profile of which exhibit significant turning maneuvers and acceleration/deceleration variations. Variations in carrier velocity were generated exclusively through the driver’s arbitrary modulation of acceleration, deceleration, and start–stop maneuvers. This approach is justified by the imperative to accumulate a training dataset replete with richly featured observations, a goal that requires frequent velocity fluctuations during the measurement campaign to adequately excite diverse dynamic responses. It should be noted, however, that the specific manner in which such velocity variations are induced is not subject to stringent prescription.

The experimentally acquired data were first processed using a low-pass filter to remove high-frequency noise. For model training, the collected data were organized into a dataset following the composition format specified in Section 5.2. A total of 7200 data points were divided into training and testing sets in a 5:1 ratio, resulting in 6000 samples for training and 1200 for testing. The training data were further normalized using Z-score standardization to mitigate the influence of varying data scales on the training performance.

### 5.2. Experiments Validation

#### 5.2.1. Experimental Conditions

Based on the preliminary experimental data, the proposed error compensation model was successfully constructed after parameter identification and model training. To validate the practical application effect and generalizability of the proposed method for dynamic error compensation of ground vehicle gravity anomaly measurement, repeated ground vehicle dynamic gravity surveys were conducted under varying kinematic conditions across three distinct routes. Acquired datasets facilitated gravity signal extraction and algorithmic verification of dynamic error compensation.

The experimental equipment for the validation experiment is identical to that used for acquiring the model training data, as detailed in Section 5.1.1. The roadmap illustrated in Figure A9 of Appendix B are experimental routes selected in Tianjin, China, with favorable road conditions, low traffic flow, and absent traffic signal interference. These gravity measurements were carried out under stable operation, obvious acceleration and deceleration operation, and high-dynamic operation, respectively. The total one-way mileage of Line 1 is 29 km. During the experiment, a constant speed of 30 km/h was maintained, and a total of two round-trip measurements were conducted. The total one-way mileage of Line 2 is 18 km. During the experiment, there was a significant acceleration and deceleration process of the carrier, with an average speed of approximately 15 km/h. A repeated measurement was conducted in one round trip. The experimental vehicle performed random high-dynamic maneuvering operations on Line 3, with a total one-way mileage of about 20 km and an average speed of approximately 60 km/h. It conducted repeated measurements in one round trip. Gravity reference values for all lines were established via the same static CG-5 gravimeter measurements followed by interpolation.

The navigation data and dynamic gravity anomaly measurements from each survey line were organized into a dataset conforming to the input specifications of the model. The collected experimental data were first processed with a low-pass filter to remove high-frequency noise. For machine learning model training, Z-score normalization was further applied to mitigate the impact of inconsistent input scales on training performance. Using the preliminary experimental data, parameter identification for the polynomial error compensation model and training of the LSTM model optimized via NAS were conducted, thereby fully constructing the proposed dynamic error compensation method for ground vehicle gravity measurement. The established method was then applied to multiple validation experiments to assess its practicality and generalization capability. Specifically, the data acquired in each validation experiment were structured according to the input requirements of the respective error compensation models. The processed outputs yielded the compensated gravity anomaly measurements. The measurement data from all survey lines were sequentially processed, and a statistical analysis was performed to evaluate the resulting accuracy.

#### 5.2.2. Determination of Algorithm Parameters

The optimization tasks addressed in this study include parameter identification for the polynomial model and architecture search for the LSTM network. The former is a 15-dimensional problem, with the fitness function guiding the evolutionary process defined as the RMSE between the model-fitted values and the ground truth. This constitutes a medium-dimensional problem of moderate complexity. In contrast, the LSTM architecture search involves 36 decision variables, and the fitness is similarly evaluated using the RMSE between model predictions and actual values. However, the computation of this metric requires frequent and computationally intensive LSTM training, rendering it a high-dimensional, highly complex optimization problem. Based on the characteristics of each problem and established practices in population-based optimization, the high-dimensional, high-complexity task was allocated a larger population size and more generations (Popsize = 300, IterNum = 1000) to ensure convergence. Additionally, MergerNum was set to 200 to enable periodic and uniform algorithm merging during the iterative process, and CENum was empirically configured as 100. For the medium-complexity parameter identification problem, optimal convergence could be achieved with lower computational cost. To balance performance and efficiency, the parameters were set as follows: Popsize = 100, IterNum = 200, MergerNum = 40, and CENum = 20. The base algorithms and other parameters of the proposed SCES remained consistent with those used in the algorithm testing phase described in Section 4.1.

As outlined in Section 5.2, the network architecture and hyperparameters of the LSTM used for machine learning-based error compensation were determined through NAS. In terms of the time step, it determines how far back the model looks to make current predictions, which is intrinsically related to the characteristic time scales of the dynamic gravity measurement process. Therefore, the time step should be determined based on domain-specific knowledge to ensure it sufficiently covers the dominant time scales of the dynamic error sources. The primary error sources in dynamic gravity measurement include short-period vibrations, long-period turning and pitching motions, and the Eötvös effect, which are closely related to the carrier’s heading, velocity, and maneuverability. These errors typically exhibit characteristic time scales ranging from 1s to 60s. To robustly capture the underlying patterns, it is generally recommended that the sequence length cover two major error cycles. Given the 1 Hz sampling rate of the gravity data, the time step was initially estimated to be 128.

#### 5.2.3. Software Environment Configuration

Due to the involvement of machine learning model training, the experimental platform is deployed on an Ubuntu 20.04 system, and the experimental environment is built based on the deep learning framework of Python 3.8. The hardware configuration is equipped with NVIDIA RTX 3090 GPUs produced by Shenzhen Rainbow Technology Development Co., Ltd., Shenzhen, China and CUDA 11.2 acceleration libraries to support large-scale LSTM model training.

### 5.3. Error Compensation Results of Proposed Method

#### 5.3.1. Error Compensation Based on Method I

Following data acquisition in the preliminary experiment, model inputs were constructed in accordance with Equation (9). Model coefficients served as optimizable individuals within the first SCES for precise parameter identification. The fitness function was defined as the RMSE between error-compensated gravity measurements and gravity reference values along the survey line. Optimal model coefficients were output upon minimization of the fitness function, yielding the polynomial error compensation model.

**Figure 7:**
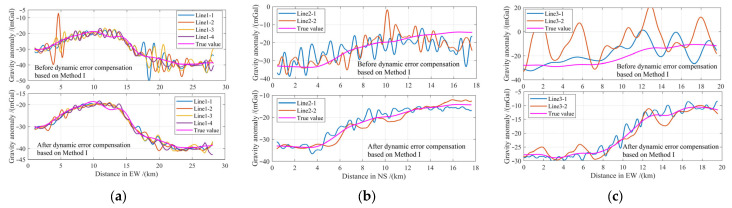
Results of before and after polynomial error model compensation on Lines 1-3. (**a**) Results of Line 1; (**b**) results of Line 2; (**c**) results of Line 3.

Formal gravity measurements and error compensation experiments share identical model inputs and error propagation relationships with the preliminary experiment. Consequently, the yielded polynomial error compensation model was directly applied to other dynamic gravity measurements, simultaneously validating its generalizability and robustness. Figure 7 presents the uncompensated measurement results and error-compensated outputs for Lines 1–3. Experimental results demonstrate that the measurements exhibit strong agreement with the ground truth, preliminarily confirming the validity of the method. However, significant dynamic errors induced by carrier maneuvers are observed, which severely degrade the measurement accuracy. This degradation is particularly pronounced under high-dynamic conditions. Following polynomial model-based error compensation (Method I), systematic and trend-related errors are effectively mitigated.

The internal/external coincidence accuracy metrics of experimental results are quantified in Table 6. Prior to error compensation, the internal and external consistency accuracies achieved on Line 1 during stable operation were moderate, reaching 2.67 mGal and 3.12 mGal, respectively. On Line 2, which involved repeated acceleration and deceleration, the corresponding accuracies of 3.25 mGal and 5.58 mGal were already unsatisfactory. More critically, under high-dynamic conditions on Line 3, the external consistency accuracy exceeded 10 mGal, rendering the measurements practically unusable for any realistic application. The internal coincidence accuracies for Lines 1-3 reach 0.85 mGal, 1.24 mGal, and 1.51 mGal, respectively, with external consistency accuracies of 1.12 mGal, 1.49 mGal, and 1.65 mGal achieved after error compensation. Nevertheless, persistent residual errors requiring further suppression remain after compensation.

#### 5.3.2. Error Compensation Based on Method II

**Figure A10:**
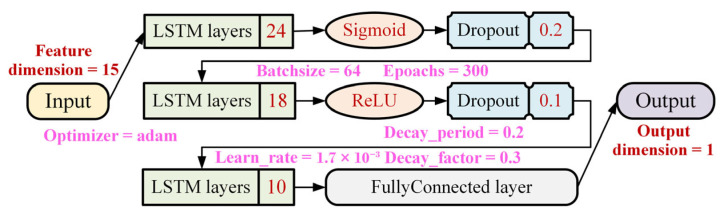
The optimal LSTM architecture of Method II obtained from NAS.

The optimal LSTM architecture obtained through NAS based on the second SCES, which is depicted in Figure A10 of Appendix B. This model accepts a 15-dimensional input and outputs the gravity measurement error. The network structure consists of three stacked LSTM layers with 24, 18, and 10 neurons, respectively. Sigmoid and ReLU activation functions follow the first and second LSTM layers, accompanied by dropout rates of 0.2 and 0.1. The final output is generated via a fully connected layer. Key hyperparameters are annotated in pink within Figure A10 of Appendix B.

**Figure 8:**
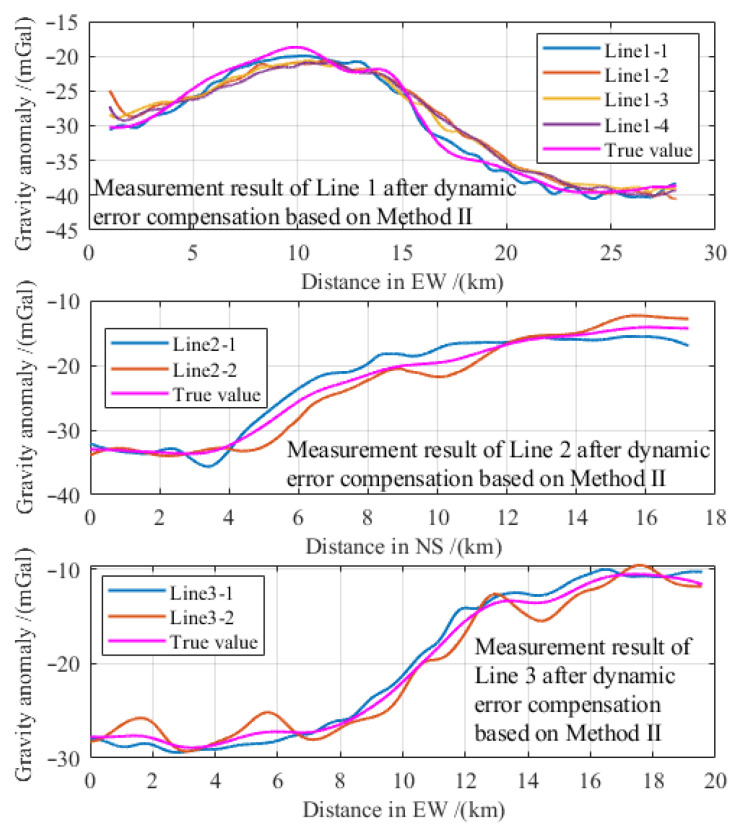
Results of LSTM model compensation on Lines 1-3.

The multi-layer LSTM model derived from Figure A10 of Appendix B was subsequently applied for error compensation in additional dynamic gravity measurement experiments. This deployment evaluated the model’s practical efficacy in dynamic error compensation while simultaneously assessing its universality and generalization. Figure 8 presents the error-compensated measurement results for Lines 1-3. Following error compensation using the optimized LSTM model derived from NAS, the complex nonlinear unmodeled errors across the three survey lines were effectively compensated for, resulting in significantly further improved measurement accuracy. The corresponding internal and external consistency accuracies are summarized in Table 6. After compensation via Method II, the internal consistency accuracies for Lines 1-3 reached 0.69 mGal, 0.84 mGal, and 1.38 mGal, respectively, while the external consistency accuracies attained 0.75 mGal, 0.89 mGal, and 1.44 mGal.

#### 5.3.3. Error Compensation Based on Method III

The polynomial and LSTM-based error compensation models address errors from distinct perspectives, with experimental results demonstrating the efficacy of both approaches. The proposed two-stage error compensation method combines these techniques, which first applies polynomial error compensation, followed by LSTM-based compensation. This strategy not only compensates for unmodeled errors unexplained by the polynomial model through machine learning but also provides theoretical grounding and interpretability for the data-driven approach. By effectively integrating physical and data-driven models, this method is particularly suited for scenarios where measurement errors contain both pronounced systematic components and complex nonlinear unmodeled elements, representing a highly effective error compensation strategy for practical engineering applications.

**Figure 9:**
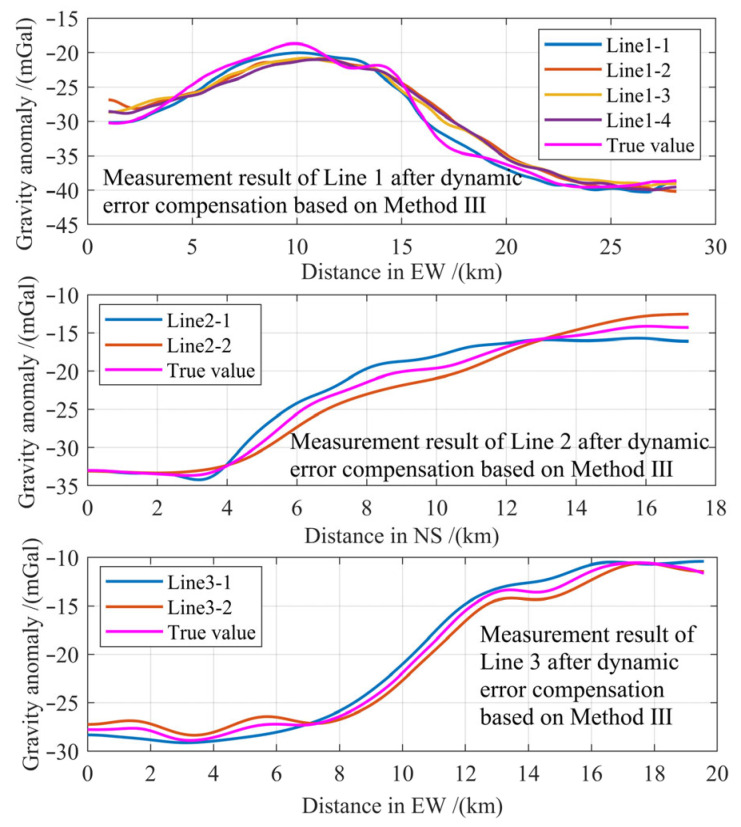
Results of two-stage error compensation model implementation on Line 1-3.

**Figure A11:**
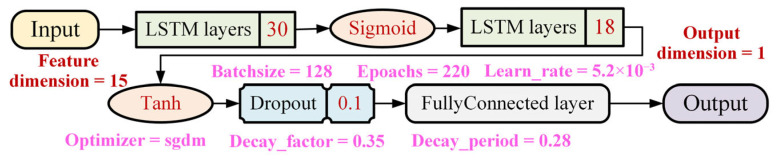
The optimal LSTM architecture of second stage in Method III.

The technical workflow of the combined two-stage error compensation model is illustrated in Figure 6. Within this workflow, the polynomial error compensation model corresponds to the description provided in Section 5.1, while the re-optimized LSTM architecture obtained through NAS specifically for residual error estimation is depicted in Figure A11 of Appendix B. Figure 9 presents gravity measurement results after error compensation for Lines 1-3, and the corresponding internal and external consistency accuracies are summarized in Table 6.

By integrating the strengths of both compensation models, Method III effectively compensates for global, trend-like systematic errors using the polynomial model, followed by targeted compensation of residual errors via the machine learning model. This approach yields breakthrough improvements surpassing any single-method compensation strategy. Following compensation via Method III, the internal consistency accuracies for Lines 1-3 reached 0.58 mGal, 0.69 mGal, and 1.14 mGal, respectively, while the external consistency accuracies attained 0.62 mGal, 0.70 mGal, and 1.15 mGal. This represents maximum average improvement of 81.15% and 86.39% in internal and external consistency, respectively. These results unequivocally demonstrate that the two-stage, hybrid error compensation method constitutes the optimal strategy for dynamic error suppression.

The excellent performance of Method III lies in its reasonable error compensation division mechanism, while Method I based on polynomial fitting can effectively compensate for global trend term errors. On this basis, the LSTM data-driven method II can avoid the learning burden of large-scale, simple trends and focus on nonlinear, stochastic residuals. This reasonable division of labor reduces the learning difficulty of LSTM, making the overall compensation process both physically interpretable and data-driven flexible, thereby achieving breakthroughs in accuracy and stability.

### 5.4. Discussion and Analysis

#### 5.4.1. Discussion and Analysis of Experimental Results

The uncompensated raw measurements yielded internal and external consistency accuracies of only 2.67 mGal and 3.12 mGal, respectively, even under stable operating conditions. This performance fails to meet the requirements for engineering application, let alone the over 10 mGal accuracy observed in highly dynamic environments. This indicates that dynamic errors induced by platform maneuvers significantly degrade measurement accuracy during dynamic gravity surveys. All three proposed error compensation methods effectively suppressed dynamic errors, substantially improving measurement accuracy. Remarkably, even in highly dynamic and complex scenarios, the worst-case compensated accuracy achieved was 1.65 mGal. This demonstrates that effective error compensation is crucial for realizing high-precision dynamic gravity measurements.

Among the three methods, the hybrid Method III, integrating the first two approaches, demonstrated superior overall compensation performance. It achieved the highest internal consistency accuracy of 0.58 mGal on the stable Line 1 and the maximum improvement percentage of 91.58% on the highly dynamic Line 3, outperforming either individual method. A detailed comparative analysis of the three methods is presented in Section 5.4.2.

Analysis of pre-compensation results reveals a pronounced amplification effect of platform dynamics on measurement errors. Internal consistency degraded from 2.67 mGal under stable conditions to 8.39 mGal under high dynamics, while external consistency surged from 3.12 mGal to 13.66 mGal. This underscores that more aggressive maneuvers induce larger errors, creating a greater imperative for compensation. After compensation, Method III delivered significantly greater improvement under high dynamics (91.58% for external consistency accuracy) compared to stable conditions (80.13%). This pattern held for the other methods, indicating higher compensation potential in dynamic environments. The fundamental reason for this phenomenon is that the error level during stable operation is relatively small, and under high-dynamic conditions, systematic errors caused by acceleration, deceleration, turning, etc., become dominant, leading to a sharp increase in measurement errors. The error compensation method proposed in this paper is good at capturing and eliminating such deterministic errors. Therefore, under high-dynamic conditions, the absolute amount of errors that can be eliminated and the corresponding relative improvement ratio are significantly larger. This indicates that the proposed method has stronger pertinence and effectiveness in solving the problem of deteriorating high-dynamic measurement accuracy.

However, despite these larger relative improvements, absolute accuracy under high dynamics remains inferior to that achieved under stable conditions. Therefore, maintaining platform stability during dynamic gravity measurements is fundamental for obtaining high-precision results.

#### 5.4.2. Comparative Analysis of Three Methods

Method I, based on the polynomial model, offers a complete theoretical framework and a solid mathematical foundation, resulting in a highly interpretable error compensation model. This approach excels at capturing global, trend-like systematic errors and is characterized by structural simplicity, computational efficiency, and ease of implementation. However, its inherent limitations in precisely fitting errors arising from highly nonlinear and complex interactions constrain its compensation effectiveness.

Method II, based on the optimal LSTM model via NAS, is distinguished by its powerful nonlinear fitting capability and pattern recognition capacity. It effectively learns complex, high-dimensional, and even implicit error patterns and residuals. However, its limitations include a substantial demand for training data and high model complexity resulting in a “black-box” nature with limited interpretability. Furthermore, relying solely on this machine learning model can lead to inconsistent performance under specific conditions, posing a risk of compensation failure, which introduces potential reliability concerns.

Method III, which integrates both models in a sequential approach, effectively combines their respective strengths. The first-stage polynomial model isolates the primary, systematic, trend-like errors. The second-stage machine learning model is then tasked solely with learning and compensating for the residual errors, which are nonlinear, stochastic, and inadequately captured components missed by the polynomial model. This focused approach allows the machine learning model to specialize in its domain of competence, preventing it from expending modeling capacity on simple trends. Experimental results confirm that this method delivers optimal error compensation performance and the most stable behavior, establishing it as an advanced strategy for enhancing the accuracy and system stability of dynamic gravity measurements.

In summary, Method I provides clear physical interpretability but has limited ability to fit complex nonlinear residuals. Method II has strong nonlinear mapping capabilities but lacks physical guidance. Method III achieves complementary advantages through a two-stage serial structure. Firstly, a mechanism model is used to efficiently handle known trend errors, and then a data model is used to fit unknown residual errors. This combination mechanism is the fundamental reason why its compensation accuracy and robustness are significantly better than any single method.

#### 5.4.3. Advantages and Limitations of the Proposed Strategy

Method III, which integrates both models, offers distinct advantages, including high compensation accuracy, robust stability, and partial interpretability. Firstly, the initial polynomial model provides a robust baseline compensation based on the proposed innovative SCES for parameter identification, ensuring a substantial reduction in the overall error level. Building upon this foundation, the machine learning model further refines the compensation by capturing and mitigating residual errors characterized by high-order nonlinearities and localized features, thereby pushing the accuracy to a higher level. Theoretically, the final compensated accuracy surpasses the limits achievable by either model used in isolation. Secondly, the polynomial base layer establishes a predictable and stable compensation baseline. Crucially, even under extreme conditions or in data-sparse regions, the polynomial model typically yields a physically plausible compensation value. While the machine learning model, focused solely on residual compensation, might exhibit instability in rare cases, its impact on the overall compensation result is constrained by the stable output of the first stage. This inherent design significantly mitigates the risk of catastrophic compensation failure for the entire scheme. Finally, and importantly, the first-stage polynomial model generally possesses a clear physical or mathematical interpretation, aiding in understanding the primary sources of system error. Although the second-stage machine learning model remains a “black box,” its responsibility is confined to compensating residuals. These residuals themselves may correspond to more specific physical mechanisms or phenomena that are inherently difficult to express with simple analytical formulations. By decomposing the error into distinct components, the hybrid two-step model ensures that at least the primary error sources remain fundamentally interpretable. Consequently, the overall error compensation model offers greater interpretability compared to purely data-driven machine learning approaches.

However, it must be acknowledged that the proposed Method III exhibits limitations regarding model complexity, computational cost, and compensation latency. Its practical implementation necessitates the selection and optimization of two distinct models. Furthermore, if the final compensation outcome proves unsatisfactory, troubleshooting and maintenance become more complex and challenging due to the increased dimensionality of the problem. Additionally, Method III requires the sequential execution of both models for compensation. This inherently incurs higher computational resource consumption than any single approach and introduces greater processing delay. Consequently, for systems demanding stringent real-time constraints, this latency may emerge as the primary bottleneck limiting their widespread adoption.

## 6. Conclusions and Prospects

Dynamic gravity measurements hold critically important and extensive engineering application value. However, dynamic errors induced by platform maneuvers severely constrain the improvement in their measurement accuracy. Consequently, research into effective dynamic error compensation methods is pivotal for acquiring high-precision gravity information and advancing the practical deployment of gravity-aided weapon systems. To address this challenge, this paper proposes three dynamic error compensation methods. (1) Polynomial model compensation based on mechanism analysis and optimized identification. Through in-depth analysis of the influence mechanisms underlying dynamic gravity measurement errors, a polynomial error compensation model was constructed. Model parameters were identified with high accuracy, stability, and reliability using a proposed novel SCES. (2) Intelligent compensation based on NAS and data-driven learning. A data-driven compensation model employing a multi-layer LSTM neural network was proposed. NAS technology was leveraged to automatically identify the optimal network architecture, effectively circumventing potential performance bottlenecks associated with manual network design. (3) Hybrid compensation fusing mechanism analysis and data-driven learning. A two-stage hybrid compensation strategy was proposed. This strategy first utilizes the polynomial model to compensate for systematic trend errors, then applies a machine learning model (LSTM) to compensate for the residual complex nonlinear errors. This approach synergistically leverages the respective strengths of mechanism-based and data-driven models.

To validate the effectiveness of the proposed methods, repeated surveys of gravity measurement and error compensation experiments were conducted along three lines with varying maneuver intensities. The experimental results demonstrate that all three proposed methods effectively compensate for dynamic errors, with the hybrid compensation strategy exhibiting the most superior overall performance. On the stably operated Line 1, an internal consistency accuracy of 0.58 mGal was achieved. Under high-dynamics conditions, the maximum accuracy improvement percentage reached 91.58%, while an external consistency accuracy of 1.15 mGal was attained. Furthermore, the enhancement in accuracy after dynamic error compensation becomes more pronounced with increasing platform maneuver intensity. However, higher absolute measurement precision is achievable in relatively stable operating environments.

The error compensation methods proposed in this study, particularly the hybrid strategy, provide effective pathways for enhancing the accuracy of dynamic gravity measurements. Nevertheless, the lightweight design of the compensation models to reduce computational load and improve real-time performance is crucial for unlocking their practical engineering utility. Additionally, exploring swarm optimization algorithms with enhanced global search capabilities and advanced machine learning models (e.g., Bi-LSTM, Transformer) capable of capturing deeper spatiotemporal dependencies represents key future research directions for achieving even more effective dynamic error compensation.

## Figures and Tables

**Table 1:** Comparison test results with cooperation fusion-related algorithms.

Algorithm	*f*_1_ (*d* = 20, object value = 300)				*f*_2_ (*d* = 20, object value = 400)				*f*_3_ (*d* = 20, object value = 600)			
	Mean	Best	Std	Rank	Mean	Best	Std	Rank	Mean	Best	Std	Rank
SCES	**300.0095**	**300.0073**	**6.660 × 10^−4^**	**1**	**400.0899**	**400.0662**	**0.006821**	**1**	**600.0095**	**600.0072**	**5.496 × 10^−4^**	**1**
LGSI	**300.1829**	**300.1248**	**4.591 × 10^−5^**	**2**	**416.1326**	**400.1039**	**25.11634**	**2**	**603.3622**	**601.2346**	**3.19243**	**2**
SSOA	2.554 × 10^11^	23,167.256	7.426 × 10^11^	5	5103.247	2360.132	1129.330	5	716.6646	690.6230	16.1928	6
CSMO	14,331.892	12,157.756	3098.0568	4	953.9027	822.7091	99.91208	4	643.5403	642.2197	3.04527	4
GA-ACO	4.479 × 10^12^	3.201 × 10^12^	4.417 × 10^11^	6	5750.318	5445.285	160.8038	6	694.3321	684.9303	3.38158	5
PSO-GWO	5592.663	313.4760	6837.785	3	611.1301	443.7236	396.0454	3	640.5684	600.2044	30.6069	3
Algorithm	*f*_4_ (*d* = 20, object value = 800)				*f*_5_ (*d* = 20, object value = 900)				*f*_6_ (*d* = 20, object value = 1800)			
	Mean	Best	Std	Rank	Mean	Best	Std	Rank	Mean	Best	Std	Rank
SCES	**867.5078**	**824.8740**	**16.9584**	**1**	**900.8525**	**900.0651**	**1.623002**	**1**	**1907.043**	**1801.628**	**157.6972**	**1**
LGSI	**876.0644**	**833.8285**	**15.1379**	**2**	**918.7728**	**900.0915**	**83.89389**	**2**	**2361.125**	**1824.475**	**902.0814**	**2**
SSOA	1027.412	970.9775	22.1996	6	5889.209	3422.955	1030.034	6	4.8234 × 10^9^	9.0237 × 10^8^	1.7021 × 10^9^	5
CSMO	946.9079	941.3430	3.43173	4	3109.112	2937.767	192.3360	4	1.2531 × 10^8^	8.7468 × 10^7^	4.7343 × 10^7^	4
GA-ACO	983.2188	953.5064	7.73833	5	4500.510	3800.955	260.7729	5	6.9691 × 10^9^	6.6233 × 10^9^	1.3108 × 10^8^	6
PSO-GWO	895.3763	849.6530	15.3696	3	2089.795	900.3343	714.9138	3	5.2549 × 10^6^	1917.537	1.8290 × 10^7^	3
Algorithm	*f*_7_ (*d* = 20, object value = 2000)				*f*_8_ (*d* = 20, object value = 2200)				*f*_9_ (*d* = 20, object value = 2300)			
	Mean	Best	Std	Rank	Mean	Best	Std	Rank	Mean	Best	Std	Rank
SCES	**2020.596**	**2001.307**	**7.675832**	**1**	**2221.128**	**2204.966**	**0.62194**	**1**	**2480.781**	**2480.781**	**4.57 × 10^−13^**	**1**
LGSI	2137.733	2098.578	16.47061	3	2247.243	2233.610	5.76406	3	**2542.865**	**2513.101**	**13.18457**	**2**
SSOA	2562.647	2255.539	98.6208	5	56,033.82	2590.333	59,584.26	6	4572.843	3204.521	760.9686	5
CSMO	**2108.287**	**2104.874**	**6.66027**	**2**	**2242.047**	**2241.111**	**2.566844**	**2**	2594.524	2582.240	22.30669	4
GA-ACO	2580.570	2527.034	21.1908	6	13,371.12	12,681.56	283.6041	5	5521.668	5295.599	70.61656	6
PSO-GWO	2283.956	2025.272	220.242	4	2369.415	2223.514	167.3910	4	2544.681	2480.793	131.9346	3
Algorithm	*f*_10_ (*d* = 20, object value = 2400)				*f*_11_ (*d* = 2, object value = 2600)				*f*_12_ (*d* = 2, object value = 2700)			
	Mean	Best	Std	Rank	Mean	Best	Std	Rank	Mean	Best	Std	Rank
SCES	**2412.351**	**2411.842**	**0.778923**	**1**	**2749.022**	**2600.005**	**57.36972**	**1**	**2960.694**	**2930.593**	**3.568205**	**2**
LGSI	**2525.504**	**2507.332**	**14.81931**	**2**	**2806.682**	**2600.473**	**150.7174**	**2**	2961.237	2943.187	13.12506	3
SSOA	7970.922	3371.769	1174.584	6	10,038.42	8816.469	393.9194	5	5621.693	4302.662	554.8651	5
CSMO	2540.666	2538.864	9.174815	3	4853.982	4639.930	346.0014	4	**2900.004**	**2900.004**	**5.451 × 10^−5^**	**1**
GA-ACO	7483.782	6867.794	245.3070	5	10,069.38	9758.464	127.1385	6	6964.645	6310.936	140.0910	6
PSO-GWO	4206.106	2500.383	1073.368	4	4093.506	2906.166	1246.313	3	3217.313	2934.027	555.1454	4

**Table 2:** Wilcoxon rank-sum test for SCES against other cooperation fusion-related algorithms.

Algorithm	*f*_1_	*f*_2_	*f*_3_	*f*_4_	*f*_5_	*f*_6_	*f*_7_	*f*_8_	*f*_9_	*f*_10_	*f*_11_	*f*_12_
LGSI	**1.15 × 10^−10^**	**8.97 × 10^−10^**	**5.73 × 10^−12^**	**3.16 × 10^−11^**	**2.91 × 10^−5^**	**1.01 × 10^−10^**	**2.37 × 10^−12^**	**1.72 × 10^−12^**	**2.36 × 10^−11^**	**3.02 × 10^−11^**	1.15 × 10^−1^	**3.18 × 10^−5^**
SSOA	**1.72 × 10^−12^**	**1.95 × 10^−11^**	**3.15 × 10^−11^**	**2.75 × 10^−12^**	**2.73 × 10^−11^**	**3.02 × 10^−11^**	**3.02 × 10^−11^**	**1.12 × 10^−11^**	**2.55 × 10^−12^**	**1.19 × 10^−12^**	**2.33 × 10^−11^**	**1.95 × 10^−11^**
CSMO	**3.02 × 10^−11^**	**3.69 × 10^−11^**	**3.02 × 10^−11^**	**1.19 × 10^−10^**	**1.09 × 10^−11^**	**3.02 × 10^−11^**	**4.18 × 10^−9^**	**1.02 × 10^−11^**	**1.39 × 10^−7^**	**8.15 × 10^−12^**	2.31 × 10^−1^	**2.88 × 10^−10^**
GA-ACO	**1.72 × 10^−12^**	**1.01 × 10^−10^**	**5.73 × 10^−12^**	**1.93 × 10^−11^**	**1.21 × 10^−10^**	**2.37 × 10^−12^**	**3.02 × 10^−11^**	**8.15 × 10^−11^**	**5.09 × 10^−8^**	**7.12 × 10^−12^**	**1.18 × 10^−7^**	5.52 × 10^−1^
PSO-GWO	**1.65 × 10^−11^**	**1.17 × 10^−11^**	**1.72 × 10^−12^**	**4.25 × 10^−10^**	**5.58 × 10^−9^**	**2.25 × 10^−11^**	**3.02 × 10^−11^**	**5.45 × 10^−11^**	**3.11 × 10^−12^**	**2.17 × 10^−11^**	**3.01 × 10^−9^**	1.25 × 10^−1^

**Table 3:** Comparison test results with single algorithm used independently.

Algorithm	*f*_1_ (*d* = 20, object value = 300)				*f*_2_ (*d* = 20, object value = 400)				*f*_3_ (*d* = 20, object value = 600)			
	Mean	Best	Std	Rank	Mean	Best	Std	Rank	Mean	Best	Std	Rank
SCES	**300.0095**	**300.0073**	**6.660 × 10^−4^**	**1**	**400.0899**	**400.0662**	**0.006821**	**1**	**600.0095**	**600.0072**	**5.496 × 10^−4^**	**1**
SCSO	5072.434	439.0727	2795.548	10	572.5940	564.6159	7.946752	11	662.0382	618.7985	11.20041	12
SSA	300.0784	300.0701	0.008987	5	449.0845	449.0845	9.25 × 10^−11^	8	651.7411	621.1431	12.07512	10
SWO	300.0775	300.0700	0.006595	4	**414.0627**	**400.1126**	**25.72711**	**3**	611.6890	601.5350	9.349197	6
PSO	300.0907	300.0091	8.782 × 10^−4^	6	623.9894	600.1540	29.96443	12	**600.0937**	**600.0607**	**3.928 × 10^−3^**	**3**
KOA	**300.0570**	**300.0220**	**0.022243**	**3**	449.0845	449.0845	1.122 × 10^−13^	7	634.0648	621.2367	7.027520	9
GWO	1.7415 × 10^3^	1.3482 × 10^3^	1.6915 × 10^3^	9	417.2514	402.3254	14.20612	4	601.2436	601.6247	1.236417	5
WOA	5.1524 × 10^3^	1.5627 × 10^3^	2.8107 × 10^3^	11	417.3135	401.2319	26.03452	5	629.1645	605.1128	12.16253	8
NOA	303.0715	300.0684	2.429231	8	540.7106	475.3165	62.12172	10	600.1315	600.0952	0.010714	4
GA	300.2526	300.0817	4.134 × 10^−3^	7	**412.1254**	**400.0985**	**19.11631**	**2**	**600.0215**	**600.0432**	**0.016523**	**2**
FATA	2.5577 × 10^4^	1.2327 × 10^4^	6.7489 × 10^3^	12	642.0516	504.2308	89.10209	13	654.6998	631.4625	8.90014	11
LPO	2.7893 × 10^4^	1.3729 × 10^4^	9.0936 × 10^3^	13	503.2511	459.3586	34.01889	9	669.3808	630.1974	12.0435	13
BFO	**300.0212**	**300.0212**	**3.721 × 10^−14^**	**2**	424.9033	400.0018	22.98684	6	626.2986	600.0000	23.6094	7
Algorithm	*f*_4_ (*d* = 20, object value = 800)				*f*_5_ (*d* = 20, object value = 900)				*f*_6_ (*d* = 20, object value = 1800)			
	Mean	Best	Std	Rank	Mean	Best	Std	Rank	Mean	Best	Std	Rank
SCES	**867.5078**	**824.8739**	**16.9584**	**5**	**900.8525**	**900.0651**	**1.623002**	**1**	**1907.043**	**1801.628**	**157.6972**	**1**
SCSO	895.5736	870.4564	10.3380	11	2504.562	2253.356	91.99434	10	1.0160 × 10^6^	1942.604	4.2614 × 10^6^	10
SSA	892.3536	874.7003	10.1514	9	2471.664	2324.224	22.20927	9	**3371.931**	**1826.029**	**1920.618**	**3**
SWO	894.5521	881.0935	9.48668	10	2424.325	1049.082	333.0426	7	4157.686	1907.831	2264.856	7
PSO	**889.5161**	**878.6015**	**4.07772**	**8**	**915.2037**	**900.2686**	**20.70709**	**4**	8845.583	1813.864	4940.013	9
KOA	935.1449	902.2205	21.8362	13	3255.306	1955.773	435.8596	12	1.8004 × 10^7^	2.0641 × 10^6^	9.5083 × 10^6^	13
GWO	**852.1665**	**837.0949**	**9.83238**	**1**	908.0227	900.5681	12.66381	3	5702.243	1915.325	2543.241	8
WOA	859.7007	816.2671	17.3734	4	1314.842	900.0816	392.6648	6	3512.433	1827.422	1664.525	5
NOA	853.5129	821.0622	11.5297	2	921.4867	900.1540	28.35443	5	3410.417	1832.482	1876.116	4
GA	854.7021	832.1675	8.82305	3	**901.2609**	**900.1025**	**0.294922**	**2**	**2191.532**	**1812.634**	**540.2452**	**2**
FATA	924.2250	874.4656	22.5659	12	2962.845	1683.032	574.7083	11	4.8764 × 10^6^	9.8153 × 10^4^	7.4849 × 10^6^	12
LPO	883.1134	846.9752	11.3501	6	3273.939	2838.644	232.8719	13	3.6047 × 10^6^	1.0541 × 10^6^	2.0291 × 10^6^	11
BFO	887.8148	872.6318	5.07252	7	2471.153	2419.749	19.49667	8	3677.324	1855.793	2679.689	6
Algorithm	*f*_7_ (*d* = 20, object value = 2000)				*f*_8_ (*d* = 20, object value = 2200)				*f*_9_ (*d* = 20, object value = 2300)			
	Mean	Best	Std	Rank	Mean	Best	Std	Rank	Mean	Best	Std	Rank
SCES	**2020.596**	**2001.307**	**7.675832**	**1**	**2221.128**	**2204.966**	**0.62194**	**1**	**2480.781**	**2480.781**	**3.57 × 10^−13^**	**1**
SCSO	2212.243	2058.109	140.3085	10	2226.596	2226.389	0.59679	6	2525.107	2509.568	17.5204	8
SSA	2428.110	2052.156	150.4594	12	**2221.485**	**2221.482**	**0.01202**	**2**	**2480.781**	**2480.781**	**6.69 × 10^−13^**	**3**
SWO	2079.172	2033.779	33.73712	7	2234.121	2221.278	26.2495	9	2480.781	2480.781	8.91 × 10^−13^	4
PSO	**2023.468**	**2002.989**	**6.983504**	**2**	2252.665	2237.367	36.6157	11	2519.809	2514.692	7.285442	7
KOA	2175.832	2101.268	32.93959	9	2266.250	2236.141	14.1984	13	2480.781	2480.781	9.14 × 10^−13^	5
GWO	**2027.773**	**2001.851**	**10.06312**	**3**	**2223.127**	**2210.842**	**6.81562**	**3**	2561.801	2515.268	36.02441	12
WOA	2051.882	2016.642	17.74461	5	2230.538	2223.451	5.27223	7	2533.543	2464.485	13.67502	10
NOA	2072.922	2022.486	14.47361	6	2223.231	2215.356	7.62362	4	2534.183	2476.825	26.86831	11
GA	2038.894	2007.169	10.80135	4	2231.715	2224.952	6.20545	8	2529.282	2529.282	4.443 × 10^−5^	9
FATA	2213.277	2149.465	45.50218	11	2245.506	2228.477	26.53153	12	2617.452	2525.303	47.52437	13
LPO	2470.069	2118.401	105.7116	13	2252.176	2240.334	8.275517	10	2496.873	2484.788	6.455445	6
BFO	2085.496	2033.280	31.86343	8	2224.617	2220.198	6.572533	5	**2480.781**	**2480.781**	**4.26 × 10^−13^**	**2**
Algorithm	*f*_10_ (*d* = 20, object value = 2400)				*f*_11_ (*d* = 2, object value = 2600)				*f*_12_ (*d* = 2, object value = 2700)			
	Mean	Best	Std	Rank	Mean	Best	Std	Rank	Mean	Best	Std	Rank
SCES	**2412.351**	**2411.842**	**0.778923**	**1**	**2749.022**	**2600.005**	**57.36972**	**1**	**2960.694**	**2930.593**	**3.568205**	**3**
SCSO	2472.081	2444.883	9.634812	3	3666.971	2926.081	725.8747	12	3026.379	2952.431	69.55637	9
SSA	2501.214	2420.127	22.81224	8	2887.010	2600.009	194.4656	3	3149.015	2936.964	477.3971	12
SWO	2499.087	2469.692	5.000541	5	**2869.353**	**2600.470**	**86.88362**	**2**	**3001.441**	**2958.489**	**26.67623**	**8**
PSO	**2437.192**	**2417.042**	**11.33156**	**2**	2916.667	2600.010	98.55059	7	3130.061	2943.121	458.2652	11
KOA	2496.725	2467.535	8.151233	4	2906.667	2600.009	94.44117	6	3030.892	2981.999	27.65031	10
GWO	2565.873	2421.223	55.98113	11	2919.953	2783.081	136.8724	8	2998.250	2945.675	34.93003	7
WOA	2542.295	2420.812	64.97248	10	2927.381	2900.000	44.70893	9	2969.934	2942.266	20.40426	4
NOA	2510.799	2421.362	74.64133	9	**2893.338**	**2600.010**	**86.82122**	**4**	**2941.473**	**2933.749**	**3.342951**	**1**
GA	**2500.211**	**2415.672**	**0.041274**	**6**	2894.936	2600.953	378.4185	5	2942.696	2934.462	3.602094	2
FATA	4453.007	2570.387	1140.166	13	4010.912	3136.106	584.3947	13	3258.298	3023.923	107.4952	13
LPO	3057.155	2501.016	1108.196	12	3465.949	3304.823	141.6872	11	2975.706	2956.593	9.576636	5
BFO	2500.419	2500.256	0.064698	7	2951.209	2600.000	91.57380	10	2976.847	2936.592	35.47161	6

**Table 4:** Wilcoxon rank-sum test for SCES against other single algorithm used independently.

Algorithm	*f*_1_	*f*_2_	*f*_3_	*f*_4_	*f*_5_	*f*_6_	*f*_7_	*f*_8_	*f*_9_	*f*_10_	*f*_11_	*f*_12_
SCSO	**3.02 × 10^−11^**	**3.02 × 10^−11^**	**1.07 × 10^−11^**	**3.02 × 10^−11^**	**1.74 × 10^−12^**	**1.76 × 10^−12^**	**3.01 × 10^−11^**	**1.68 × 10^−9^**	**2.98 × 10^−11^**	**3.02 × 10^−11^**	**2.93 × 10^−10^**	**1.72 × 10^−12^**
SSA	**1.21 × 10^−12^**	**5.57 × 10^−10^**	**3.15 × 10^−12^**	**4.31 × 10^−8^**	**9.79 × 10^−5^**	**2.22 × 10^−11^**	**1.07 × 10^−11^**	**6.52 × 10^−7^**	**4.63 × 10^−10^**	**2.99 × 10^−11^**	**1.85 × 10^−3^**	9.31 × 10^−2^
SWO	**1.32 × 10^−9^**	**1.17 × 10^9^**	**1.05 × 10^−5^**	**2.28 × 10^−6^**	**5.09 × 10^−11^**	**1.73 × 10^−11^**	**4.95 × 10^−11^**	**3.74 × 10^−7^**	**2.38 × 10^−8^**	**6.72 × 10^−10^**	1.95 × 10^−1^	**3.37 × 10^−11^**
PSO	**1.72 × 10^−12^**	**1.23 × 10^−11^**	**3.15 × 10^−9^**	**2.68 × 10^−8^**	**7.73 × 10^−9^**	**3.02 × 10^−11^**	**3.02 × 10^−11^**	**7.59 × 10^−9^**	**2.83 × 10^−11^**	**1.35 × 10^−11^**	**2.01 × 10^−10^**	**7.12 × 10^−11^**
KOA	**1.12 × 10^−12^**	**3.15 × 10^−11^**	**4.56 × 10^−11^**	**9.06 × 10^−8^**	**1.07 × 10^−11^**	**8.99 × 10^−11^**	**3.02 × 10^−11^**	**2.99 × 10^−11^**	**4.09 × 10^−12^**	1.26 × 10^−1^	**5.78 × 10^−8^**	**3.02 × 10^−11^**
GWO	**3.02 × 10^−11^**	**3.02 × 10^−11^**	**1.07 × 10^−11^**	**3.02 × 10^−11^**	**3.02 × 10^−11^**	**3.02 × 10^−11^**	**1.72 × 10^−12^**	**4.63 × 10^−10^**	**5.57 × 10^−10^**	**4.56 × 10^−9^**	**1.17 × 10^−9^**	**1.40 × 10^−10^**
WOA	**4.68 × 10^−11^**	**1.92 × 10^−11^**	**2.64 × 10^−10^**	**1.15 × 10^−8^**	**4.31 × 10^−5^**	**2.35 × 10^−10^**	**6.21 × 10^−3^**	**2.93 × 10^−10^**	**3.33 × 10^−10^**	**2.96 × 10^−10^**	**9.53 × 10^−3^**	**1.85 × 10^−3^**
NOA	**1.72 × 10^−12^**	**1.12 × 10^−12^**	**3.02 × 10^−11^**	**1.93 × 10^−12^**	**4.48 × 10^−11^**	**4.10 × 10^−11^**	**6.01 × 10^−8^**	**2.21 × 10^−11^**	**3.15 × 10^−11^**	**1.35 × 10^−10^**	**7.84 × 10^−9^**	**2.93 × 10^−10^**
GA	**3.02 × 10^−11^**	**3.02 × 10^−11^**	**1.59 × 10^−11^**	**2.94 × 10^−10^**	**5.78 × 10^−8^**	**1.72 × 10^−9^**	**1.26 × 10^−11^**	**1.43 × 10^−5^**	**1.97 × 10^−2^**	**4.15 × 10^−8^**	**2.35 × 10^−10^**	**2.68 × 10^−10^**
FATA	**2.98 × 10^−8^**	**4.09 × 10^−12^**	**4.69 × 10^−11^**	**2.65 × 10^−10^**	**5.78 × 10^−9^**	**4.50 × 10^−10^**	**3.02 × 10^−11^**	**3.34 × 10^−11^**	**1.58 × 10^−10^**	**3.69 × 10^−10^**	9.11 × 10^−1^	**1.15 × 10^−2^**
LPO	**3.15 × 10^−10^**	**1.12 × 10^−9^**	**8.89 × 10^−10^**	**4.11 × 10^−11^**	**2.02 × 10^−8^**	**1.72 × 10^−11^**	**3.15 × 10^−9^**	**5.09 × 10^−6^**	**4.31 × 10^−11^**	**1.73 × 10^−6^**	7.96 × 10^−2^	**6.03 × 10^−9^**
BBO	**1.72 × 10^−12^**	**3.02 × 10^−11^**	**1.18 × 10^−10^**	**3.15 × 10^−10^**	**9.06 × 10^−8^**	**2.02 × 10^−8^**	**5.09 × 10^−11^**	**4.63 × 10^−11^**	3.87 × 10^−1^	**2.11 × 10^−10^**	**3.48 × 10^−11^**	**2.39 × 10^−10^**

**Table 5:** Instrument parameters of main equipment.

Sensors	Parameters	Accuracy
SINS	Sample rate	100 Hz
	Gyro drift-bias	0.003°/h
	Gyro random walk	0.0003°/$\sqrt{h}$
	Acc zero-bias stability	20 μg/day
	Acc random walk	5 $\mu g/\sqrt{Hz}$
odometer	Counting error	1 × 10^−4^
	Mileage error	1 m/10 km
barometer	Resolution	5 Pa
	Height error	1 m
GNSS	Sample rate	10 Hz
	Positioning error	0.01 m (RMS)
	Velocity measurement error	0.02 m/s (RMS)
CG-5	Resolution	1 μGal
	Repeatability accuracy	5 μGal
	Long-term drift	0.05 mGal/day

**Table 6:** Accuracy statistics of results before and after error compensation. (mGal).

Conditions	Coincidence Accuracy	Before Compensation	After Compensation Based on Method I (Percentage)	After Compensation Based on Method II (Percentage)	After Compensation Based on Method III (Percentage)
Line 1	Internal	2.67	0.85 (68.16%)	0.69 (74.16%)	0.58 (78.28%)
	External	3.12	1.12 (64.10%)	0.75 (75.96%)	0.62 (80.13%)
Line 2	Internal	3.25	1.24 (61.85%)	0.84 (74.15%)	0.69 (78.77%)
	External	5.58	1.49 (73.30%)	0.89 (84.05%)	0.70 (87.46%)
Line 3	Internal	8.39	1.51 (82.00%)	1.38 (83.55%)	1.14 (86.41%)
	External	13.66	1.65 (87.92%)	1.44 (89.46%)	1.15 (91.58%)

## Data Availability

The data presented in this study are available on request from the corresponding author.

## DURC Statement

Current research is limited to gravity dynamic measurement and error compensation 1434 technology, which is extremely beneficial for engineering infrastructure construction, earthquake 1435 disaster warning, basic scientific research, resource exploration, autonomous navigation, 1436 unmanned driving and other technological research, and does not pose a threat to public health 1437 and national security. As an ethical responsibility, authors strictly adhere to relevant national and 1438 international laws about DURC. Authors advocate for responsible deployment, ethical 1439 considerations, regulatory compliance, and transparent reporting to mitigate misuse risks and 1440 foster beneficial outcomes.

## Abbreviations

The following abbreviations are used in this manuscript:

SCES	Swarm Cooperation Evolution Strategy
LSTM	Long Short-Term Memory
NAS	Neural Architecture Search
GNSS	Global Navigation Satellite System
GA	Genetic Algorithm
PSO	Particle Swarm Optimization
GWO	Grey Wolf Optimizer
SCSO	Sand Cat Swarm Optimization
SSA	Sparrow Search Algorithm
LPO	Lung Performance Optimizer
BFO	Bitterling Fish Optimization
RNN	Recurrent Neural Networks
SWO	Spider Wasp Optimization
KOA	Kepler Optimization Algorithm
LGSI	Ludo Game Swarm Intelligence
SSOA	Synergistic Swarm Optimization Algorithm
CSMO	Co-evolution Spider Monkey Optimization
ACO	Ant Colony Optimization
OE	Overall Effectiveness
BR	Best Reachability
WOA	Whale Optimization Algorithm
NOA	Nutcracker Optimization Algorithm
SINS	Strapdown Inertial Navigation System
OD	Odometer

## Appendix A

**Table A1:** Details of CEC2022 test functions.

Function Set	No.	Functions	Category	Range	Dimension	Best Value
CEC2022	*f*_1_	Shifted and full Rotated Zakharov Function	Unimodal	[−100, 100]	20	300
	*f*_2_	Shifted and full Rotated Rosenbrock’s Function	Simple Multimodal	[−100, 100]	20	400
	*f*_3_	Shifted and full Rotated Expanded Schaffer’s Function		[−100, 100]	20	600
	*f*_4_	Shifted and full Rotated Non-Continuous Rastrigin’s Function		[−100, 100]	20	800
	*f*_5_	Shifted and full Rotated Levy Function		[−100, 100]	20	900
	*f*_6_	Hybrid Function 1 (N = 3)	Hybrid	[−100, 100]	20	1800
	*f*_7_	Hybrid Function 2 (N = 6)		[−100, 100]	20	2000
	*f*_8_	Hybrid Function 3 (N = 5)		[−100, 100]	20	2200
	*f*_9_	Composition Function 1 (N = 5)	Composition	[−100, 100]	20	2300
	*f*_10_	Composition Function 2 (N = 4)		[−100, 100]	20	2400
	*f*_11_	Composition Function 3 (N = 5)		[−100, 100]	20	2600
	*f*_12_	Composition Function 4 (N = 6)		[−100, 100]	20	2700
